# Mechanistic characterization of UDP‐glucuronic acid 4‐epimerase

**DOI:** 10.1111/febs.15478

**Published:** 2020-08-05

**Authors:** Annika J. E. Borg, Alexander Dennig, Hansjörg Weber, Bernd Nidetzky

**Affiliations:** ^1^ Institute of Biotechnology and Biochemical Engineering Graz University of Technology NAWI Graz Austria; ^2^ Austrian Centre of Industrial Biotechnology Graz Austria; ^3^ Institute of Organic Chemistry Graz University of Technology NAWI Graz Austria

**Keywords:** decarboxylase, epimerase, kinetic isotope effect, short‐chain dehydrogenase/reductase, UDP‐glucuronic acid

## Abstract

UDP‐glucuronic acid (UDP‐GlcA) is a central precursor in sugar nucleotide biosynthesis and common substrate for C4‐epimerases and decarboxylases releasing UDP‐galacturonic acid (UDP‐GalA) and UDP‐pentose products, respectively. Despite the different reactions catalyzed, the enzymes are believed to share mechanistic analogy rooted in their joint membership to the short‐chain dehydrogenase/reductase (SDR) protein superfamily: Oxidation at the substrate C4 by enzyme‐bound NAD^+^ initiates the catalytic pathway. Here, we present mechanistic characterization of the C4‐epimerization of UDP‐GlcA, which in comparison with the corresponding decarboxylation has been largely unexplored. The UDP‐GlcA 4‐epimerase from *Bacillus cereus* functions as a homodimer and contains one NAD^+^/subunit (*k*_cat_ = 0.25 ± 0.01 s^−1^). The epimerization of UDP‐GlcA proceeds via hydride transfer from and to the substrate’s C4 while retaining the enzyme‐bound cofactor in its oxidized form (≥ 97%) at steady state and without trace of decarboxylation. The *k*_cat_ for UDP‐GlcA conversion shows a kinetic isotope effect of 2.0 (±0.1) derived from substrate deuteration at C4. The proposed enzymatic mechanism involves a transient UDP‐4‐keto‐hexose‐uronic acid intermediate whose formation is rate‐limiting overall, and is governed by a conformational step before hydride abstraction from UDP‐GlcA. Precise positioning of the substrate in a kinetically slow binding step may be important for the epimerase to establish stereo‐electronic constraints in which decarboxylation of the labile β‐keto acid species is prevented effectively. Mutagenesis and pH studies implicate the conserved Tyr149 as the catalytic base for substrate oxidation and show its involvement in the substrate positioning step. Collectively, this study suggests that based on overall mechanistic analogy, stereo‐electronic control may be a distinguishing feature of catalysis by SDR‐type epimerases and decarboxylases active on UDP‐GlcA.

## Graphical abstract

UDP‐glucuronic acid 4‐epimerase is a short‐chain dehydrogenase/reductase that uses tightly bound NAD^+^ to catalyze interconversion of UDP‐glucuronic acid and UDP‐galacturonic acid. Here, the authors present a biochemical mechanistic study of the enzyme from *Bacillus cereus*. Epimerization involves oxidation at the substrate C4 and nonstereoselective reduction in a 4‐keto intermediate. Substrate positioning in a kinetically slow binding step can establish stereo‐electronic constraints to prevent decarboxylation of the intermediate.

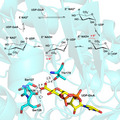

**Abbreviations**
KIE: kinetic isotope effect
SDR: short‐chain dehydrogenase/reductase
UGAepi: UDP‐glucuronic acid 4‐epimerase

## Introduction

**Fig. 1:**
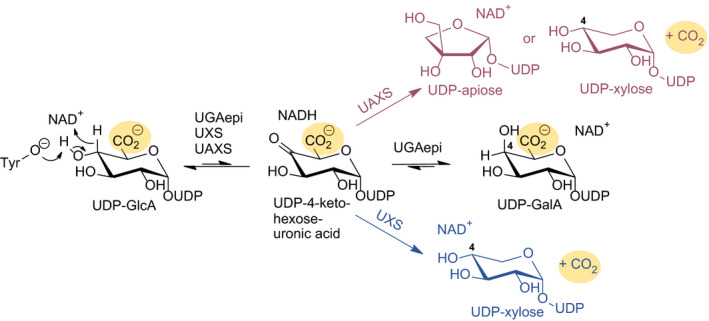
Simplified reaction pathways for UDP‐apiose/xylose synthase (UAXS), UDP‐xylose synthase (UXS), and UDP‐glucuronic acid 4‐epimerase (UGAepi). The routes leading to decarboxylation are highlighted in red (UAXS) and blue (UXS), and position of CO_2_ is assigned with a yellow circle. Orientation of the C4‐hydroxyl group in the products is equatorial in case of UDP‐xylose (UAXS, UXS) and axial in UDP‐GalA (UGAepi). UDP‐GlcA, uridine diphosphate glucuronic acid; UDP‐GalA, uridine diphosphate galacturonic acid.

Uridine 5′‐diphosphate α‐d‐glucuronic acid (UDP‐GlcA) is a central intermediate at the cross‐roads of diverse metabolisms, including the biosynthesis of essential monosaccharides (e.g., d‐xylose) [1] and polysaccharides (e.g., pectin, alginate) [2], the biosynthesis of secondary metabolites [3], and detoxification [4, 5]. In plants and microorganisms, UDP‐GlcA is the immediate precursor of three important monosaccharides (d‐galacturonic acid, d‐xylose, and d‐apiose) required in cell wall polysaccharide biosynthesis [6, 7]. Besides their importance in cell biology, the sugar nucleotide syntheses from UDP‐GlcA are of considerable interest for mechanistic enzyme research. Three enzymes (UDP‐GlcA epimerase, UGAepi, EC 5.1.3.6; UDP‐xylose synthase, UXS, EC 4.1.1.35; and UDP‐apiose/xylose synthase, UAXS, EC 4.1.1.) share a common substrate in order to form distinct products from it [8, 9, 10]. The enzymes are evolutionary related by common membership to the short‐chain dehydrogenase/reductase (SDR) protein superfamily [11]. All belong to the extended subclass of SDRs that are distinct from the canonical SDR type in having NAD^+^ coenzyme tightly bound to their structure [11, 12, 13]. Common feature of their mechanism appears to be a transient UDP‐4‐keto‐hexose‐uronic acid intermediate which is formed by proton abstraction from C4‐OH together with hydride transfer to enzyme‐NAD^+^ [13, 14, 15, 16, 17, 18, 19]. At this stage, the catalytic paths of the individual enzymes diverge (Fig. 1) [11]. Remarkably, only UGAepi avoids decarboxylation of UDP‐GlcA. Further, the enzyme is able to catalyze re‐addition of the hydride at the equatorial position of the C4 to achieve the D‐glucose to D‐galactose switch in stereochemistry [20]. Little is known about determinants of the finely tuned reactivity in each of these enzymes. While the reactions of UDP‐xylose synthase (UXS) and UDP‐apiose/xylose synthase (UAXS) have been characterized mechanistically [13, 17, 21], the one of UDP‐GlcA epimerase (UGAepi) is yet unexplored for direct evidence in support of the central claims of enzymatic mechanism.

A transient UDP‐4‐keto‐hexose‐uronic acid species in the UGAepi reaction is currently supported solely by inference to the UXS and UDP‐galactose 4‐epimerase (GALE) reactions [13, 16, 22]. GALE, active on UDP‐glucose (UDP‐Glc), is mechanistically the best‐characterized member of the extended SDR family. GALE promotes a 180°‐degree rotation of the formed 4‐ketopyranose intermediate around the phosphate backbone, therefore offering the opposite face of the sugar for reduction by NADH [16, 23]. A similar rotation has been assumed, although not shown, to be involved in UGAepi reactions as well [22]. The central role of a highly conserved Tyr as the catalytic base for initiating the oxidation at C4 of the sugar has been proposed for UGAepi, based on the similarity to the GALE reaction [22, 24, 25]. UGAepi has been described from plants and bacteria in studies spanning several decades, with reported difficulties in recombinant production [10, 20, 22, 26, 27, 28]. The enzymes were often found to be quite unstable in isolated form, thus complicating their detailed characterization.

Here, we report the identification of a new UGAepi from *Bacillus cereus HuA2‐4* (BcUGAepi). The enzyme was conveniently produced in *Escherichia coli* (30 mg·L^−1^) and proved quite robust during isolation, storage, and use. Thus, it was a practical candidate for the proposed mechanistic study. We performed here a comprehensive biochemical and steady‐state kinetic analysis of the enzyme. We developed efficient enzymatic syntheses of UDP‐GalA and UDP‐4‐[^2^H]‐GlcA as substrate for the reverse reaction of the epimerase and as mechanistic probe, respectively. Using UDP‐4‐[^2^H]‐GlcA and *in situ* real‐time monitoring of the reaction by proton NMR, we demonstrated that the overall net epimerization involves two stereospecific steps of hydride transfer, from and to the sugar C4, and proceeds without loss of deuterium label from the substrate in the product. We further used site‐directed mutagenesis, kinetic isotope effects, and pH studies to obtain deepened insight into the enzyme mechanism. We provide evidence supporting catalytic reaction via a transient UDP‐4‐keto‐hexose‐uronic acid intermediate. Reduction in the intermediate is considerably faster than its formation. A kinetic isomerization of the enzyme–substrate complex before the hydride abstraction from UDP‐GlcA is rate‐limiting. The isomerization step arguably reflects a slow protein conformational change in which the reactive groups on substrate and NAD^+^ are aligned with the catalytic groups on the enzyme. Based on structural comparisons and mechanistic considerations, we put forth the idea that stereo‐electronic control is crucial in the epimerase reaction to protect the labile β‐keto acid species from undergoing decarboxylation. Our study thus shows basic mechanistic analogy between SDR‐type epimerases (UGAepi) and decarboxylases (UXS, AXS) active on UDP‐GlcA and proposes essential features of their mechanistic distinction.

## Results

### Identification and biochemical characterization of a new UGAepi

BLAST search against the known microbial UGAepis revealed an uncharacterized protein (UniProt: J8BY31_BACCE) in *Bacillus cereus HuA2‐4* that was annotated as UDP‐glucose 4‐epimerase. Since the protein was similar to already known UGAepi enzymes, we wished to examine its reactivity. Protein harboring C‐terminal Strep‐tag (referred to as BcUGAepi throughout) was conveniently obtained from standard *E. coli* culture in a yield of ~30 mg purified protein per L (Fig. S1). We showed with gel filtration that the isolated protein was a homodimer of ~37‐kDa subunits (Figs S2 and S3). Absorbance spectrum indicated the presence of a protein‐bound nicotinamide cofactor, as expected (Fig. S4). Using sensitive HPLC assay for detection (Fig. S5), we identified the cofactor as NAD, not NADP. We determined that the NAD was mainly oxidized (NAD^+^). NADH was also present, the content varying slightly (4.3 ± 1.1%; *N* = 3) dependent on the enzyme batch used (Fig. S5). The protein was stable during storage (−20°C; ≥52 weeks) and under all assay conditions (pH 7.6; 23 °C) used later.

**Fig. 2:**
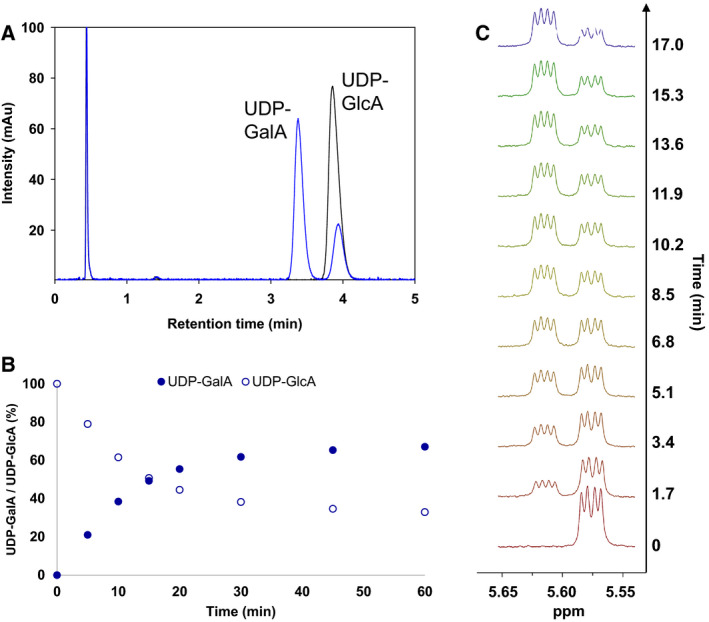
Analysis of the reaction of BcUGAepi. (A) Achiral HPLC analysis of the reaction mixture of BcUGAepi with UDP‐GlcA as substrate (sample at equilibrium state) is shown in blue. HPLC chromatogram of a control reaction (without enzyme) is shown in black. (B) Time course of BcUGAepi reaction with UDP‐GlcA as a substrate. The reaction mixture contained 1 mm UDP‐GlcA, 100 µm NAD^+^, and 2 µm (0.07 mg·mL^−1^) purified recombinant BcUGAepi in sodium phosphate buffer (50 mm Na_2_HPO_4_, 100 mm NaCl, pH 7.6). (C) Snapshot of *in situ* ^1^H‐NMR of the BcUGAepi reaction (in D_2_O) with UDP‐GlcA as a substrate (0–16.8 min). Signals from the anomeric proton of UDP‐GlcA (5.57 ppm) and UDP‐GalA (5.62 ppm) are shown. UDP‐GlcA, uridine diphosphate glucuronic acid; UDP‐GalA, uridine diphosphate galacturonic acid.

We then offered sugar nucleotides (1 mm; UDP‐Glc, UDP‐GlcA, UDP‐Xyl) as substrates of the putative epimerase (2 µm) and monitored their conversion with HPLC. UDP‐Glc and UDP‐Xyl were not reactive above the detection limit (~5 µm), irrespective of the addition of NAD^+^ (up to 1 mm) to the reaction. UDP‐GlcA was a substrate, and it was converted into a single product detectable by HPLC (Fig. 2A). Using authentic standard of UDP‐GalA synthesized according to herein developed procedure (see later), we confirmed the identity of the product and thus demonstrated the enzymatic reactivity as epimerization at C4 of UDP‐GlcA.

We show in Fig. 2B the results of time‐course study of conversion of UDP‐GlcA. The reaction proceeded to equilibrium whose position (*K*_eq_ = [UDP‐GalA]/[UDP‐GlcA]) we determined as 2.0 ± 0.1 (*N* = 10). We also showed that NAD^+^ (0–1000 µm) had no effect on the reaction rate nor on the equilibrium position (Fig. S6), indicating that the cofactor is tightly bound to the enzyme during the recombinant expression. A specific activity for UDP‐GlcA epimerization of 0.6 U·mg^−1^ was calculated from the data. The enzyme activity was unaffected by pH in the range 7.0–9.0. It increased with temperature up to 37 °C (Fig. S7). However, to avoid convoluted effects of temperature on enzyme and sugar nucleotide stability, we chose 23 °C for further experiments. Besides demonstrating the enzymatic reactivity, our results also illuminate the substrate specificity of the epimerase, showing that a carboxylate group at C5 of the UDP‐sugar is required for enzymatic function.

We further used proton NMR to monitor the epimerization directly from the enzymatic reaction mixture in D_2_O solvent (pD 7.6). The conversion of UDP‐GlcA into UDP‐GalA was conveniently recorded from the change in the corresponding anomeric proton signals, as shown in Fig. 2C. The equilibrium ratio of UDP‐GalA and UDP‐GlcA was ~2, consistent with the results obtained in H_2_O. The NMR data confirmed the results from HPLC analysis that UDP‐GalA was the sole product of the enzymatic reaction. Release of an intermediate, or of a potential side product resulting from adventitious decarboxylation, was ruled out at level of approximately the enzyme molarity used (~5 µm; HPLC).

### Enzymatic synthesis of UDP‐GalA and UDP‐4‐[^2^H]‐GlcA

To characterize UGAepi kinetically, we required UDP‐GalA as enzyme substrate for the reverse direction of reaction. We considered previous protocol that had obtained UDP‐GalA from commercial α‐galacturonic acid 1‐phosphate, using enzymatic reaction with UTP [29]. Since α‐galacturonic acid 1‐phosphate is no longer offered, we attempted its synthesis by anomeric phosphorylation of GalA but were unsuccessful for lack of a suited kinase. In our hands, the GalA kinase [30] suffered from poor stability in its isolated form and showed insufficient activity with GalA. We considered isolation of UDP‐GalA from an epimerase reaction mixture at equilibrium; however, preparative HPLC separation of UDP‐GalA from UDP‐GlcA proved impractical due to the low yield (~1 mg) obtained. We improved on the isolation efficiency by exploiting the enzyme ArnA for selective conversion of UDP‐4‐ketoxylose (UDP‐β‐l‐threo‐pentopyranosid‐4‐ulose; (Scheme S1) [31, 32]. The ArnA does not utilize UDP‐GalA within limits of detection. The UDP‐GalA is conveniently separated from UDP‐4‐keto‐xylose, thus enabling its isolation in high purity (≥ 98%) and in amounts of 10–50 mg (Figs S8 and S9). The identity of UDP‐GalA was confirmed by HPLC and NMR (Figs S10 and S11).

The UDP‐4‐[^2^H]‐GlcA was prepared as mechanistic probe to analyze hydrogen transfer steps of the overall C4 epimerization of UDP‐GlcA. The synthetic route was effective that reported earlier [33, 34], except that regeneration of NAD^+^ for twofold oxidation of UDP‐glucose was done using the pyruvate/L‐lactate dehydrogenase system [35]. This seemingly small change in procedure was, however, crucial to enhance the efficiency of the key oxidative step catalyzed by UDP‐glucose dehydrogenase (Scheme S2). The efficient NAD^+^ regeneration system also allowed higher substrate concentration (up to 30 mm) while retaining full conversion to the desired product. UDP‐4‐[^2^H]‐GlcA was obtained in high purity (≥ 99.5%) and in amounts of 40–50 mg (Figs S12 and S13). The yield from 4‐[^2^H]‐glucose was ~50%. The identity of UDP‐4‐[^2^H]‐GlcA was confirmed by HPLC and NMR (Figs S14 and S15). The [^2^H] content at C4 was 99% or greater.

### Kinetic characterization of UGAepi

Initial rates of C4 epimerization were recorded using UDP‐GlcA and UDP‐GalA as the substrate. Time courses of substrate consumption were linear in the early phase of the reaction (Fig. 2b, Fig. S16), and there was a closed balance between substrate utilized and product formed at all conditions used. Kinetic parameters (*k*_cat_, *K*_m_) were obtained from nonlinear fits of regular hyperbolic dependencies of the initial rate upon the substrate concentration, as shown in Fig. S17. The *K*_m_ was 0.36 ± 0.02 mm for UDP‐GlcA and 0.89 ± 0.13 mm for UDP‐GalA. The *k*_cat_ was 0.25 ± 0.01 s^−1^ for UDP‐GlcA and 0.32 ± 0.02 for s^−1^ for UDP‐GalA. The catalytic efficiencies (*k*_cat_/*K*_m_) for forward (0.71 mm ^−1^·s^−1^) epimerization and reverse (0.35 mm ^−1^ s^−1^) epimerization are used to calculate the *K*_eq_ from the Haldane relationship for the reaction [36]. The kinetically determined *K*_eq_ of 2.0 is in excellent agreement with the equilibrium constant measured directly. Kinetic parameters for the forward epimerization agree between this epimerase and other such enzymes of microbial and plant origin [20, 22, 26, 27]. However, this is the first full kinetic study of a UDP‐GlcA 4‐epimerase at steady state.

### pH studies of wild‐type enzyme and Y149F variant

**Fig. 3:**
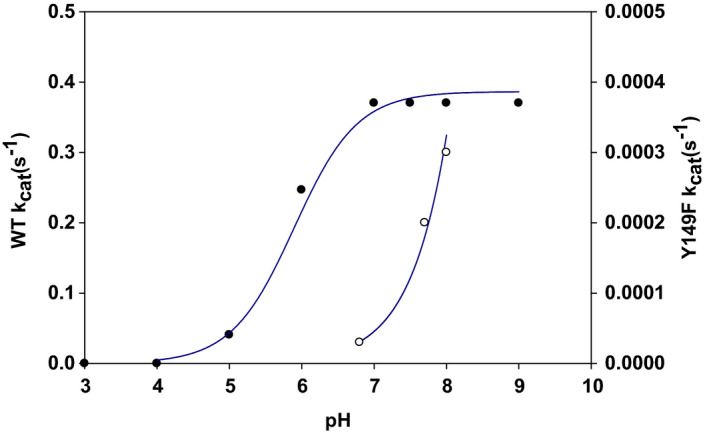
Apparent *k*_cat_ values for the BcUGAepi reaction with UDP‐GlcA dependent on pH. Symbols show the data, and the solid line is the corresponding fit. Closed circles correspond to wild‐type and open circles to Y149F variant of BcUGAepi.

The canonical active site of SDR enzymes involves an invariant tyrosine whose canonical role in catalysis is that of a general base to facilitate alcohol oxidation by NAD^+^ [11]. Sequence comparison with the well‐studied UDP‐Gal 4‐epimerase revealed Tyr149 as the candidate residue in UGAepi (Fig. S18). We used replacement with phenylalanine to disrupt the catalytic functionality of the original tyrosine. The purified Y149F variant (Fig. S19) contained protein‐bound NAD^+^ as the wild‐type enzyme. As anticipated from the proposed role of Tyr149 in catalysis, the Y149F variant was highly impaired in epimerase activity that was about 1.2 × 10^3^‐fold lower than the wild‐type activity (Fig. S20). Nonetheless, the Y149F variant converted UDP‐GlcA into UDP‐GalA without trace formation of side products (e.g., due to decarboxylation) or intermediates. Note: we rigorously excluded contamination by the wild‐type enzyme during purification of the Y149F variant (see Materials and methods). In addition, the triplet codon exchange (TAC → TTC) used for mutagenesis made translational errors during incorporation of Phe149 into the Y149F variant extremely unlikely [37, 38, 39, 40]. Generally, removal of base catalytic assistance to the reaction does not, by necessity, lead to a complete loss of activity in the variant enzyme. Other factors effective in catalysis (e.g., positioning) might suffice to reduce the energetic barrier such that reaction can still occur at a detectable rate [41]. To further address the question of acid–base catalysis in UGAepi, we carried out pH studies with wild‐type enzyme and Y149F variant. Results are shown in Fig. 3.

The pH dependence of the wild‐type enzyme was sigmoidal with low and high levels of activity (*k*_cat_) at low and high pH, respectively. Model for a mechanism with a single ionizable group that must be unprotonated for enzyme activity was used to fit the data [42]. An apparent p*K*_a_ of 5.90 (±0.05) was calculated. The Y149F variant was inactive at pH 6.5 or lower. Activity was found at pH ~7 and the logarithmic *k*_cat_ increased linearly with pH, showing a slope of 0.85 (±0.07). These results suggested that the Y149F variant might exploit alternative forms of base catalysis, perhaps from H_2_O/OH^‐^ or from another enzyme residue, as proposed for an analogous tyrosine‐to‐phenylalanine variant of GALE [24, 43]. They provide strong evidence in support of a catalytic base function of Tyr^149^ in the wild‐type UGAepi.

### Isotope‐labeling studies and kinetic isotope effects

To explore the epimerization mechanism used by the enzyme, we analyzed with *in situ* NMR the conversion of UDP‐4‐[^2^H]‐GlcA catalyzed by wild‐type UGAepi. We show in Fig. S21 that deuterium label at the C4 of substrate was retained completely at the C4 of UDP‐GalA product. The overall epimerization is thus shown to consist of two stereospecific steps of hydrogen transfer: abstraction (to NAD^+^) of the axial C4 hydrogen/deuterium in the d‐*gluco* configuration and transfer back to C4 (from NADH) in an equatorial position to establish the d‐*galacto* configuration. The reaction proceeds through a transient UDP‐4‐keto‐hexose‐uronic acid intermediate that is not released from the enzyme.

Having determined that epimerization involves cleavage and formation of C4‐H bond, we analyzed the effect of deuterium labeling at C4 on the enzymatic reaction rates of wild‐type UGAepi and Y149F variant. We showed that the apparent *k*_cat_ for the wild‐type reaction with UDP‐4‐[^2^H]‐GlcA was 2.0 (±0.1)‐fold (*N* = 5) slower than the *k*_cat_ for the corresponding reaction with unlabeled substrate (prepared identically as the isotopically labeled one). This demonstrates that the reaction is subject to a normal primary kinetic isotope effect (KIE). The KIE (= *k*_cat_^H^/*k*_cat_^D^) was independent of pH in the range 6.0–9.5. At pH 5.0, the KIE was 3.5 (±0.3). The corresponding KIE for reaction of the Y149F variant was dependent on pH and increased from a value of 1.17 (±0.01) at pH 7.0 to higher values of 2.39 (±0.09) at pH 8.0 and 3.53 (±0.02) at pH 9.0.

The relative rates of the enzymatic half‐reactions leading to and from the UDP‐4‐keto‐hexose‐uronic acid intermediate determine the reduced‐state portion of the nicotinamide cofactor at steady state. Absorbance and fluorescence are useful to detect the enzyme‐bound NADH, but the data are challenging to quantify. We therefore measured directly with a rapid‐quenching HPLC assay the relative NADH content in the wild‐type enzyme while reacting with UDP‐GlcA or UDP‐4‐[^2^H]‐GlcA. The NADH content normalized on the total amount of cofactor present was 0.014 and 0.016 in reactions with unlabeled and labeled substrates, respectively (Figs S22 and S23). It was not different within limits of error from the relative NADH content in the enzyme as isolated (0.031) (Fig. S5).

## Discussion

### Functional annotation of UDP‐GlcA epimerases

Phylogenetic analysis places UDP‐GlcA epimerases into a distinct subgroup within the sugar nucleotide epimerase family of SDR enzymes (Fig. S24). The UDP‐pyranose 4‐epimerases (GALE, UDP‐GlcNAc 4‐epimerase) [44, 45, 46] and CDP‐D‐paratose 2‐epimerases [47, 48] constitute further subgroups of that family. The UDP‐GlcA epimerases are separated from UDP‐GlcA decarboxylases (UXS, UAXS, ArnA) that represent a different SDR family. They are also clearly separated from two‐site sugar nucleotide epimerases such as GDP‐D‐mannose 3,5‐epimerase (Fig. S24) [49] as well as from sugar nucleotide dehydratases (not shown). The UDP‐GlcA epimerases are further subdivided according to phylogenetic origin and specificity. Besides the main 4‐epimerase found in plant and bacterial subgroups, a second bacterial subgroup additionally contains 5‐epimerase that converts UDP‐GlcA into UDP‐l‐IdoA [50]. However, a recent computational analysis suggested that these 5‐epimerases might in fact all be UDP‐GlcA 4‐epimerases [51]. Studies of UGAepi substrate specificity show that the C5 carboxylate group of UDP‐GlcA is essential for substrate‐binding recognition and/or catalysis. UDP‐Glc, UDP‐Gal, UDP‐GlcNAc, and UDP‐Xyl are often not accepted as substrates by UGAepis [20, 26, 27] or give strongly decreased activity [22].

Although basic functional attributes are assignable from the sequence‐based categorization, specific correlations between molecular structure and enzymatic reactivity remain elusive in the absence of detailed mechanistic–kinetic analysis. Except for the Tyr/Ser/Lys catalytic triad of residues that are universally conserved, the residues involved in sugar binding are highly variable. There is effectively very little conservation in UGAepi of the relevant sugar‐binding residues of UDP‐pyranose 4‐epimerases [45, 52], as presented with an example of GALE in Fig. S25. The epimerase and the decarboxylase reaction both face the challenge of having to combine specificity in substrate recognition with flexibility in the positioning of the respective 4‐keto intermediate (Fig. 1). Each mechanism additionally requires that the different chemical steps of the enzymatic reaction are precisely coordinated with at least one physical step of reorientation/repositioning of the 4‐keto intermediate. Particular task of UGAepi is to handle the highly reactive UDP‐4‐keto‐hexose‐uronic acid species for epimerization which in the corresponding decarboxylases is immediately taken further to the UDP‐4‐keto‐pentulose intermediate via decarboxylation [13, 14, 17]. Only little can be inferred from the well‐characterized epimerases (GALE) and UDP‐GlcA decarboxylases (UXS, AXS) as to the particular catalytic strategy employed by UGAepi, thus necessitating the current study.

### Kinetic properties of UGAepi

**Fig. 4:**
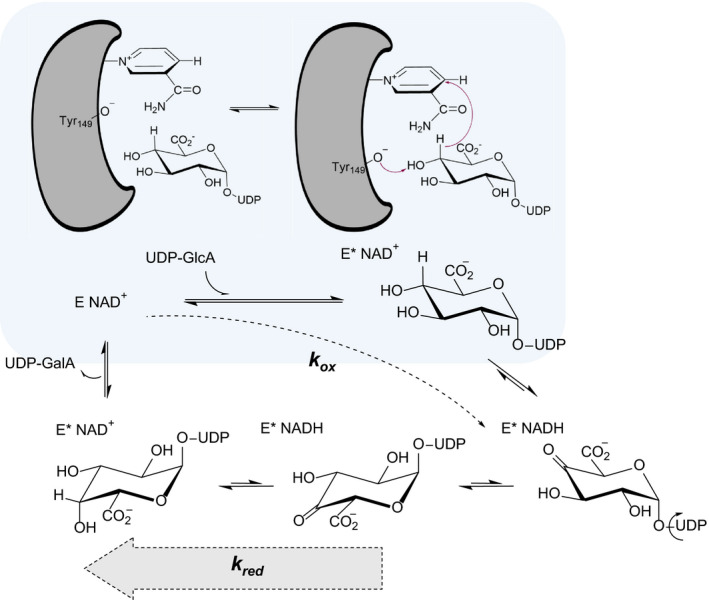
Proposed kinetic mechanism of UGAepi. The first step is the binding of uridine diphosphate glucuronic acid (UDP‐GlcA) into the free enzyme (E‐NAD^+^) including a slow conformational change (highlighted with gray background) in the enzyme to E*‐NAD^+^. Hydride transfer from C4‐H to NAD^+^ yields E*‐NADH and UDP‐4‐keto‐hexose‐uronic acid as a reaction intermediate. This 4‐keto intermediate rotates around the phosphate backbone of the substrate positioning the opposite face of the 4‐keto group toward NADH. Hydride transfer from NADH yields the product uridine diphosphate galacturonic acid (UDP‐GalA) which is released from the enzyme regenerating E‐NAD^+^. The flux through the reduction (*k*_red_) of the 4‐keto intermediate to UDP‐GalA is considerably higher than the net flux through the steps involved in the oxidation (*k*_ox_), which the thickness of arrows should illustrate. One can estimate that, for just 3% or less of total enzyme accumulated as enzyme‐NADH at the steady state when UDP‐GlcA is saturating, the flux from enzyme‐NADH to give enzyme‐NAD^+^ (*k*_red_) must exceed by two magnitude orders (≥ 30‐fold) and the net flux from enzyme‐NAD^+^ to enzyme‐NADH (*k*_ox_). The portion of enzyme‐NADH is given by the relationship *k*_ox_/(*k*_red_ + *k*_ox_).

Despite their overall structural similarity and mechanistic analogy, UGAepi and GALE show interesting differences in the observable kinetic behavior. The proposed reaction pathway for UGAepi is shown in Fig. 4.

This pathway may be largely identical with that of GALE [46] but differs from it in respect to the kinetic significance of the reductive half‐reaction, leading from the 4‐keto intermediate to the d‐*galacto* configured product. The GALE reaction with UDP‐glucose was shown to involve 19% enzyme‐NADH of total enzyme, hence enzyme‐bound 4‐keto intermediate, at the steady state [53, 54]. This indicates that both half‐reactions of the GALE mechanism proceed at rates of the same magnitude order. The absence of enzyme‐NADH in the UGAepi reaction with UDP‐GlcA shows that effectively no 4‐keto intermediate is accumulated on the enzyme at the steady state.

Two kinetic scenarios are consistent with enzyme‐NADH below detection in the catalytic reaction: (a) The overall sequence of steps leading to enzyme‐NADH is rate‐limiting. (b) Release of the UDP‐GalA product from enzyme‐NAD^+^ is rate‐limiting. The KIE of 2.0 is in accordance with the first scenario, while it refutes the second for which no isotope effect (KIE = 1) is expected. UGAepi reaction with hydride transfer to NAD^+^ as the rate‐limiting step is expected to show a sizeable KIE on *k*_cat_ (≥ 3.5), substantially larger than the one observed [55, 56, 57]. Plausible explanation is that the enzymatic mechanism involves a slow physical step, most likely a conformational rearrangement of the enzyme/NAD^+^/UDP‐GlcA complex (Fig. 4), that precedes the hydride transfer step; this physical step suppresses the intrinsic KIE on the hydride transfer step to the one effectively measurable in *k*_cat_. The proposed precatalytic step is further supported by evidence from pH studies of wild‐type enzyme and Y149F variant. At low pH, where the *k*_cat_ of wild‐type UGAepi decreases, the KIE increases to a value of 3.5 (pH 5.0). This result suggests that the hydride transfer step of the enzymatic mechanism requires a group of enzyme/NAD^+^/UDP‐GlcA to be unprotonated and therefore is dependent upon the pH. It further suggests that an additional pH‐independent step (the proposed conformational rearrangement) partly limits the *k*_cat_ at the pH optimum. The effect of substitution of Tyr149 by Phe on the pH dependencies of the *k*_cat_ and its associated KIE suggests that Tyr149 is part of the ionizable molecular group (apparent p*K*_a_ = 5.9) critical for the hydride transfer; the tyrosine is also involved in the precatalytic physical step whose probable role is to align precisely the reactive groups in enzyme/NAD^+^/UDP‐GlcA complex (Fig. 4). By analogy with the reaction of GALE, the conformational rearrangement can also be important to increase the redox potential of the enzyme‐bound NAD^+^ through favorable change in electrostatic environment [58, 59]. We note furthermore that pH studies of GALE have also pointed to an unusually low p*K*_a_ (= 6.9) for the catalytic tyrosine [43].

### UGAepi: stereo‐electronic control for catalytic epimerization under prevention of decarboxylation

**Fig. 5:**
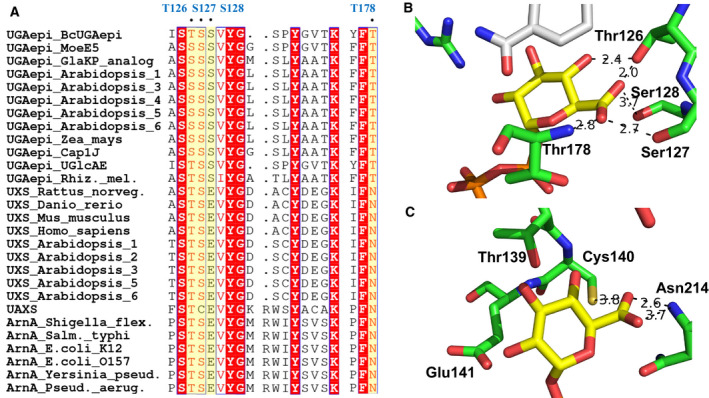
Structural comparison of UDP‐glucuronic acid 4‐epimerases (UGAepis) and SDR‐type decarboxylases active on UDP‐GlcA. ArnA, UDP‐glucuronic acid decarboxylase; UXS, UDP‐xylose synthase; UAXS, UDP‐apiose/xylose synthase. (A) Partial multiple sequence alignment (aligned with Clustal Omega and visualized with ESPript). The epimerase residues involved in binding of the carboxylate group of UDP‐GlcA are highlighted in yellow, and the corresponding residues of BcUGAepi are labeled in blue at the top of the alignment. UniProt entries: UGAepi_BcUGAepi (J8BY31), UGAepi_MoeE5 (A0A003), UGAepi_GlaKP_analog (Q9RP53), UGAepi_Arabidopsis_1 (Q9M0B6), UGAepi_Arabidopsis_3 (O81312), UGAepi_Arabidopsis_4 (O22141), UGAepi_Arabidopsis_5 (Q9STI6), UGAepi_Arabidopsis_6 (Q9LIS3), UGAepi_Zea_mays (Q304Y2), UGAepi_Cap1J (P96481), UGAepi_UGlcAE (A7GQD3), UGAepi_Rhiz._mel. (O54067), UXS_Rattus_norveg. (Q5PQX0), UXS_Danio_rerio (Q6GMI9), UXS_Mus_musculus (Q91XL3), UXS_Homo_sapiens (Q8NBZ7), UXS_Arabidopsis_1 (Q8VZC0), UXS_Arabidopsis_2 (Q9LZI2), UXS_Arabidopsis_3 (Q9FIE8), UXS_Arabidopsis_5 (Q9SN95), UXS_Arabidopsis_6 (Q9ZV36), UAXS (Q9ZUY6), ArnA_Shigella_flex. (Q83QT8), ArnA_Salm._typhi (O52325), ArnA_E.coli_K12 (P77398), ArnA_E.coli_O157 (Q8XDZ3), ArnA_Yersinia_pseud. (Q93PD8), and ArnA_Pseud._aerug. (Q9HY63). (B) Active‐site close up of BcUGAepi modeled with YASARA from the experimental UDP‐GlcA complex structure of the epimerase MoeE5 [28]. (C) Active‐site close up of the UDP‐GlcA complex structure of *Arabidopsis thaliana* UAXS (C100A variant; PDB: 6H0P), showing the relevant residues from the alignment. PyMOL was used to generate the structures.

How then does UGAepi prevent decarboxylation of the labile β‐keto acid moiety of the transient 4‐keto intermediate? The combined evidence from kinetic analysis and structural comparison (discussed below) suggests an important role for stereo‐electronic control in enzyme catalysis to the epimerization of UDP‐GlcA (for general case, see ref. 60). Using structure‐based, multiple sequence alignment (Fig. 5A), we show that residues involved in binding of the substrate’s carboxylate group differ between UGAepis and UDP‐GlcA decarboxylases (UXS, UAXS, ArnA). Crystal structure of MoeE5 [28] provides the essential structural basis for UGAepi, and a homology model of BcUGAepi shows excellent agreement with the experimental template (Fig. 5B). In UGAepi, the substrate carboxylate is embedded in a hydrogen bond network of four coordinating residues, namely Thr126, Ser127, Ser128, and Thr178 (main chain). In the decarboxylases (UAXS, Fig. 5C; UXS, Fig. S26a; ArnA, Fig. S26b), the carboxylate group appears to be more loosely bound. The overall structure of the binding pocket is changed to provide fewer interactions (compare Fig. 5C to Fig. 5B); a binding residue (Ser128) of the epimerase is replaced by an acidic residue (Glu; Fig. 5a), known from earlier studies of UXS and UAXS to play an important role in the decarboxylase catalytic mechanism. In the epimerase, the threonine from the SDR catalytic triad (Thr126 in BcUGAepi) is in hydrogen bond distance to both the C5 carboxylate and the reactive 4‐hydroxy group of the substrate. By way of comparison (Fig. S27), the 6‐OH of UDP‐Glc in *E. coli* GALE has just two coordinating residues (Asn179 and Tyr299) and is oriented away from Ser124 (the positional/functional homologue of Thr126 in BcUGAepi). The unique substrate‐binding mode in UGAepi gives rise to the suggestion that the carboxylate of UDP‐GlcA not only serves as a recognition site for binding, but it can also have an immediate involvement in substrate positioning for the catalytic event.

**Fig. 6:**
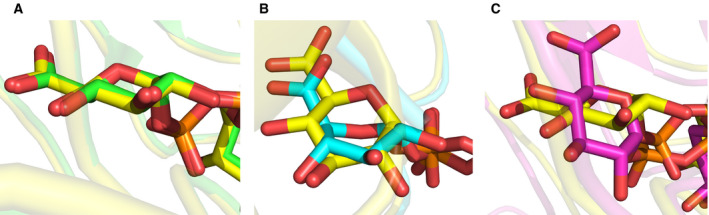
PyMOL representation of the position of the C5 carboxylate group of UDP‐GlcA bound to UGAepi, UXS, and UAXS. The yellow carbon atoms show BcUGAepi (modeled). (A) Overlay of the structures of MoeE5 (green; PDB: 6KV9) and BcUGAepi (modeled). The carboxylate group is equatorial. (B) Overlay of the structures of UXS (cyan; PDB: 2B69, sugar modeled into the active site [13]) and BcUGAepi. The axially oriented carboxylate can be seen in the structure of UXS. (C) Overlay of the structures of UAXS (pink; PDB: 6H0P) and BcUGAepi, showing the nearly equatorial carboxylate group in UAXS.

Analyzing the epimerase‐bound conformation of UDP‐GlcA in more detail, we notice that the pyranose moiety adopts an undistorted ^4^ *C*_1_ ring pucker and has the carboxylate group in a perfectly equatorial position, as shown in Fig. 6A. Based on stereo‐electronic considerations [61, 62, 63, 64], decarboxylation of the 4‐keto intermediate would be optimal with the carboxylate group‐oriented axially, for the dihedral angle between the C = O bond of the ketone and the C5‐C6 bond could thus approach the ideal ~90°. It follows that, by maintaining the dihedral angle at close to ~0° due to the equatorial carboxylate, the epimerase might constrain its catalytic reaction so as to minimize decarboxylation. To achieve this, the epimerase has to limit sugar ring distortion in its active site, which is noteworthy in light of chemical studies showing that distortion from chair conformation influences the reactivity of 2‐oxocyclohexane carboxylic acid isomers for decarboxylation in solution [61, 62]. The appealing idea of differential stereo‐electronic control in epimerase and decarboxylase reactions receives additional support from mechanistic studies of UXS and UAXS. In UXS, as revealed by molecular dynamics simulations, the enzyme‐bound UDP‐GlcA features a binding energy‐driven sugar ring distortion (^4^ *C*_1_ → *B*_O,3_) that forces the carboxylate group into an almost fully axial position (Fig. 6B) [13]. Thus, the UXS Michaelis complex provides optimal stereo‐electronic conditions for immediate decarboxylation subsequent to the oxidation at C4. The UAXS reaction exploits substrate oxidation at C4 to promote retro‐aldol ring opening between C2 and C3, and the decarboxylation happens only after the ring opening has occurred [17]. Stereo‐electronic conditions in the active site of UAXS meet requirements for the particular timing of the catalytic steps in the enzymatic conversion. The Michaelis complex involves a largely undistorted UDP‐GlcA substrate that has the carboxylate in a closely equatorial position (Fig. 6C). As suggested from molecular dynamics simulation, the ring‐opened conformation of the 4‐keto intermediate will only change later during the reaction to move the carboxylate into a quasi‐axial position suitable for decarboxylation [17]. The special importance of stereo‐electronic control for 4‐keto intermediate protection in the UGAepi reaction is emphasized by the catalytic behavior of active‐site variants of UXS and UAXS which despite showing only trace activity lack the ability to uncouple substrate oxidation at the C4 from decarboxylation at C5 [13, 14, 17].

Central task of catalysis by SDR‐type epimerases is to position their keto intermediates flexibly for nonstereospecific reduction. Studies of GALE show that the requirement can be realized through a relatively weak binding of the intermediate’s sugar moiety, compared with binding of its UDP moiety [25]. Therefore, mechanistic conundrum posed for UGAepi is to establish proper balance between stereo‐electronic control, due to a relatively more constrained substrate binding compared with GALE (compare Fig. 5B and Fig. S27), and free rotation of the 4‐keto‐hexose intermediate. It can be significant, therefore, that in terms of their *k*_cat_ the known UGAepis (this work, 0.25 s^−1^; *B. cereus* isozyme, 3.2 s^−1^, [20]; *Arabidopsis thaliana*, 24 s^−1^, [27]) are much slower enzymes than GALE (760 s^−1^_,_ [24]). Added binding interactions with the UDP‐sugar substrate in UGAepi compared with GALE may be responsible for the slowing down of the catalytic reaction. Figure 4 shows the particular distribution of fluxes across the reaction pathway of BcUGAepi. The conformational restraint imposed by the enzyme on the rate of 4‐keto intermediate formation (which also limits the *k*_cat_) might reflect the structural requirements of the active site to implement the proposed stereo‐electronic control. Rotation of the 4‐keto‐hexose‐uronic acid intermediate bound to enzyme‐NADH is part of the sequence of microscopic steps involved in the reduction of the 4‐keto intermediate to UDP‐GalA (*k*_red_ in Fig. 4). Although considerably faster than the enzyme’s *k*_cat_, the rotation step in BcUGAepi could still be appreciably slower than the analogous rotation step is in GALE. To keep a tight 'grip' on the equatorial C5 carboxylate group during a comparably slow rotation may be important for BcUGAepi to retain stereo‐electronic control during conversion of the 4‐keto intermediate into UDP‐GalA.

In summary, this study provides biochemical foundation for enzymatic C4 epimerization in UDP‐GlcA. The proposed catalytic mechanism involves a transient UDP‐4‐keto‐hexose‐uronic acid intermediate whose formation and conversion via hydride transfer to and from enzyme‐bound nicotinamide coenzyme are facilitated by Tyr149 acting as the general base/acid, respectively. While largely analogous to the GALE mechanism in its base features, the UGAepi mechanism is special in requiring the handling of a highly decarboxylation‐prone 4‐keto intermediate. Our study suggests stereo‐electronic control as the catalytic strategy developed by UDP‐GlcA 4‐epimerase to achieve this task. Precisely orchestrated structural dynamics will be essential in their implementation. Importantly, stereo‐electronic control provides an essential mechanistic distinction between UDP‐GlcA 4‐epimerase and SDR‐type decarboxylases using UDP‐GlcA as the substrate.

## Materials and methods

### Materials

The synthetic genes of BcUGAepi and ArnA were ordered in pET17b expression vector (pET17b_BcUGAepi; pET17b_ArnA) from GenScript (Piscataway, NJ, USA). Uridine monophosphate (UMP, 98% purity) was from Carbosynth (Compton, UK). Uridine diphosphate (UDP), UDP‐d‐glucuronic acid (UDP‐GlcA; > 98% purity), and sodium pyruvate was from Sigma‐Aldrich (Vienna, Austria). NAD^+^ (> 98% purity) was from Roth (Karlsruhe, Germany). Deuterium oxide (99.96% ^2^ *H*) was from Euriso‐Top (Saint‐Aubin Cedex, France). All other reagents and chemicals were of highest available purity. For plasmid DNA isolation, the GeneJET Plasmid Miniprep Kit (Thermo Scientific, Waltham, MA, USA) was used. DpnI and Q5^®^ High‐Fidelity DNA polymerase were from New England Biolabs (Frankfurt am Main, Germany). Hexokinase and d‐lactate dehydrogenase were from Megazyme (Vienna, Austria). All other enzymes were prepared in‐house. Oligonucleotide primers were from Sigma‐Aldrich. *E. coli* NEB5α competent cells were from New England Biolabs. *E. coli* Lemo21 (DE3) cells were prepared in‐house.

### Enzymes

The site‐directed substitution Tyr149 by Phe was introduced by a modified QuikChange protocol (see Supporting information for details). Both BcUGAepi wild‐type and Y149F variants were produced by expression in *E. coli* Lemo21 (DE3) cells that harbored the pET17b expression vector containing the gene of interest. The proteins were purified utilizing the C‐terminal Strep‐tag, and the size and purity of the proteins were confirmed by SDS/PAGE. For purification of the Y149F variant, new Strep‐tag columns were used to avoid any possible contamination by the wild‐type enzyme. Full details of the expression and purification conditions used are given in the Supporting information. ArnA was obtained by expression in *E. coli* BL21 (DE3) harboring the pET17b_ArnA vector using the same expression and purification procedure as for BcUGAepi.

Human UDP‐glucose 6‐dehydrogenase (hUGDH) was expressed in *E. coli* Rosetta 2 (DE3) cells harboring the vector pBEN_SGC_hUGDH [65]. Inorganic pyrophosphatase (iPPase) was produced by expression in *E. coli* BL21 (DE3) cells carrying pET‐STREP3_iPPase vector. UDP‐glucose pyrophosphorylase (UGPase) was expressed in *E. coli* BL21 (DE3) Gold cells harboring pET30_UGPase plasmid. hUGDH and iPPase were purified by affinity chromatography using Strep‐tag following the same procedure as for BcUGAepi, and UGPase was isolated by His‐tag purification (see the Supporting information for details). The activity of iPPase was determined as described in the literature [66], and the activity measurement of UGPase is described in the Supporting information.

### Substrate synthesis

#### UDP‐α‐d‐galacturonic acid

The reaction mixture (20 mL) contained 4.6 mm UDP‐GlcA (60 mg, 0.09 mmol), 1 mm NAD^+^ (13.3 mg, 0.02 mmol), and 2.7 µm BcUGAepi (0.1 mg·mL^−1^, 2 mg) in buffer (50 mm Na_2_HPO_4_, 100 mm NaCl, pH 7.6). Incubation was at 30 °C until UDP‐GalA and UDP‐GlcA were present at the equilibrium ratio of 2:1, respectively, confirmed by HPLC. BcUGAepi was removed by ultrafiltration (Vivaspin Turbo centrifugal filter tube, 30 kDa cutoff). The remaining UDP‐GlcA was converted to UDP‐α‐d‐4‐keto‐xylose (Scheme S1) by adding the following components into the reaction mixture: 10 mm sodium pyruvate (22 mg, 0.2 mmol), 20 U·mL^−1^ LDH (400 U), and 4.7 µm ArnA (0.2 mg mL^−1^, 2.8 mg). The reaction mixture was incubated at 30 °C for 16 h until all UDP‐GlcA was consumed. ArnA and LDH were removed from the mixture by ultrafiltration (30 kDa cutoff).

#### UDP‐α‐d‐4‐^2^H‐glucuronic acid

The synthesis strategy is indicated in Scheme S2. The reaction mixture (37 mL) contained 15 mm 4‐^2^H‐α‐d‐glucose (100 mg, 0.55 mmol), 50 mm uridine 5′‐triphosphate (UTP, 716.5 mg, 1.3 mmol), 5 mm MgCl_2_ (17.6 mg, 0.18 mmol), 0.13% (w/v) bovine serum albumin (BSA, 48.1 mg), and 10 µm α‐d‐glucose 1,6‐bisphosphate dissolved in 50 mm Tris/HCl buffer (pH 7.5). Hexokinase (13.8 U·mL^−1^, 510.6 U), 6.8 U·mL^−1^ phosphoglucomutase (251.6 U), 0.2 mg·mL^−1^ UGPase (7.4 mg), and 0.077 mg·mL^−1^ iPPase (2.8 mg) were added, and the reaction mixture was incubated at 30 °C for 20 h. After confirming the completeness of the reaction on HPLC, the final oxidation step toward UDP‐4‐^2^H‐GlcA was initiated by the addition of 2 mm NAD^+^ (49 mg, 0.074 mmol), 40 mm sodium pyruvate (163 mg, 1.48 mmol), 20 U·mL^−1^ LDH (740 U), and 1.23 mg·mL^−1^ hUGDH (45.5 mg), and the reaction was incubated at 37 °C for 24 h. The enzymes were removed with Vivaspin Turbo centrifugal filter tubes (10 kDa cutoff, Sartorius) prior to column purification of the nucleotide sugar.

#### Product isolation

Both UDP‐α‐d‐galacturonic acid and UDP‐α‐d‐4‐^2^H‐glucuronic acid were isolated by two‐column chromatographic purification steps. First, the desired product was separated from the other components using ÄKTA FPLC system (GE Healthcare, Vienna, Austria) connected to a 125 mL TOYOPEARL SuperQ‐650M anion exchange column (GE Healthcare) and a 10 mL sample loop. 20 mm sodium acetate solution (pH 4.3) was used as binding buffer. Compounds bound to the column were eluted with a step‐wise gradient of 1 m sodium acetate buffer at pH 4.3; see SI for details. Fractions containing the desired product were detected via UV absorption (ʎ = 254 nm), identified by HPLC, and combined prior to concentrating under reduced pressure on a Laborota 4000 rotary evaporator (Heidolph, Schwabach, Germany) at 40 °C to a final volume of approximately 5–10 mL. NaOAc was removed from the concentrated product preparation using an ÄKTA FPLC connected to Superdex G‐10 size‐exclusion column (GE Healthcare) and 5 mL sample loop. The product was detected based on its UV absorption at 254 nm and eluted with deionized water. The product‐containing fractions were combined and concentrated under reduced pressure at 40 °C to a final volume of approximately 20 mL. Residual H_2_O was removed by lyophilization (Christ Alpha 1‐4 Lyophilizer, B. Braun Biotech International, Melsungen, Germany), and the pure nucleotide sugar obtained as white powder. The purity and identity of UDP‐α‐d‐galacturonic acid and UDP‐α‐d‐4‐^2^H‐glucuronic acid were determined on HPLC and ^1^H‐NMR.

### Determination of enzyme‐bound coenzyme

The dimeric oligomeric state of BcUGAepi wild‐type was determined by gel filtration chromatography (see Supporting information for details).

#### Enzyme‐bound NAD^+^/ NADH

For studying the occupancy of BcUGAepi by NADH, the enzyme (50 µL, 18 mg·mL^−1^) was denatured by incubating with 50 µL of methanol for 3 h at room temperature. The enzyme precipitation was removed by centrifugation (16 100 ***g***, 80 min, 4 °C) and the supernatant analyzed on HPLC. The amount of released NADH was calculated based on a calibration curve (Fig. S22), where defined concentrations of NADH were prepared in double‐distilled water and directly used for measurements. UV absorbance at 262 nm was used for the detection of NAD^+^ and NADH. The occupancy of NADH in BcUGAepi was calculated in % from the concentration of released NADH (in µm) divided by the total protein concentration (in µm) used in the experiment.

#### Rapid‐quench procedure to analyze enzyme‐bound NADH from reaction

Reaction mixture containing 1 mm UDP‐GlcA or UDP‐4‐^2^H‐GlcA and 1 µm (0.035 mg·mL^−1^) of purified BcUGAepi was prepared in 10 mL of sodium phosphate buffer (50 mm Na_2_HPO_4_, 100 mm NaCl, pH 7.6). The reaction was run for 2 min at 23 °C, and afterward, 10 mL of ice‐cold phosphate buffer (50 mm Na_2_HPO_4_, 100 mm NaCl, pH 1.3) was added to decrease the pH of the reaction mixture to 4.4 and terminate the enzymatic activity, while retaining the enzyme soluble. The reaction mixture was immediately transferred into an ice‐cold Vivaspin Turbo centrifugal filter tube (30 kDa cutoff) and centrifuged at 0 °C (2880 g) until 19 mL of the mixture had flowed through the filter. The flow‐through was analyzed on HPLC, and the enzyme was washed twice with 5 mL of ice‐cold phosphate buffer (50 mm Na_2_HPO_4_, 100 mm NaCl, pH 4.4). The flow‐through from each washing step was analyzed on HPLC to confirm that the UDP‐GlcA/UDP‐GalA ratio remained unchanged over the washing procedure. The enzyme was concentrated up to the final volume of 50 µL and denatured with 50 µL of MeOH as described in the section 'Enzyme‐bound NAD^+^/ NADH'. The NADH released from the active site of BcUGAepi was detected on HPLC. As a control for the stability of NADH, the same procedure was performed for NADH standard solution to confirm that the coenzyme was not degrading during the denaturation experiment.

### BcUGAepi activity assays

Standard BcUGAepi reaction mixtures (250 µL final volume) contained 1 mm UDP‐GlcA, 100 µm NAD^+^, and 2 µm (0.07 mg·mL^−1^) purified recombinant BcUGAepi (135 µm/5 mg·mL^−1^ for variant Y149F) in sodium phosphate buffer (50 mm Na_2_HPO_4_, 100 mm NaCl, pH 7.6). The reactions were incubated at 23 °C, quenched with methanol (50% (v/v) final concentration) at desired time points, and the precipitated enzyme removed by centrifugation (16 100 ***g***, 4 °C, 30 min) prior to HPLC analysis. The initial rates were determined from the linear part of the time course by dividing the slope of the linear regression (mm·min^−1^) by the enzyme concentration (mg·mL^−1^) giving the initial rate in µmol per (min mg protein). The apparent *k*_cat_ values (s^−1^) were calculated from the initial rate with the molecular mass of the functional enzyme monomer (wild‐type BcUGAepi: 37 003 g·mol^−1^; Y149 variant: 36 987 g·mol^−1^). For the temperature optimum study, the reactions were performed under standard conditions but in varying temperatures. For NAD^+^ concentration study, the reactions were carried out under standard conditions except that lower enzyme concentration (1 µm, 0.037 mg·mL^−1^) was used.

For the determination of the kinetic parameters, BcUGAepi (2 µm, 0.07 mg·mL^−1^) was mixed with NAD^+^ (50 µm) in 50 mm Na_2_HPO_4_ buffer (containing 100 mm NaCl, pH 7.6). Varying concentrations of substrate (UDP‐GlcA/UDP‐GalA) were added into the 100 µL reaction mixture at room temperature, and the reactions were quenched after 10‐min incubation time with 25% (v/v) acetonitrile and heated up to 95 °C for 5 min. Each reaction was carried out in duplicate. After centrifugation (16 100 ***g***, 4 °C, 30 min), the samples were analyzed on HPLC and the initial rates were plotted against the substrate concentration used. The kinetic parameters were calculated with SigmaPlot 10.0 based on fitting to the Michaelis–Menten kinetic model. The *k*_cat_ is based on the molarity of the enzyme subunit.

### *In situ* ^1^H NMR

For *in situ* NMR experiments, the reaction was carried out in D_2_O buffer (50 mm K_2_HPO_4_/KH_2_PO_4_) titrated to pD 7.6, where pD corresponds to the reading of pH meter + 0.4. The reaction mixture contained 2.7 mm UDP‐GlcA (or UDP‐4‐^2^H‐GlcA), 91 µm NAD^+^, and 2.4 µm (0.09 mg·mL^−1^) purified recombinant BcUGAepi. The acquisition was carried out on a Varian INOVA 500‐MHz NMR spectrometer (Agilent Technologies, Santa Clara, CA, USA) every 30 s from the enzyme addition. The VNMRJ 2.2D software was used for the measurements. ^1^H‐NMR spectra (499.98 MHz) were recorded on a 5‐mm indirect detection PFG probe with presaturation of the water signal by a shaped pulse. The spectra were analyzed using MestReNova 16.0 (Mestrelab Research, S.L., Santiago de Compostela, Spain).

### pH studies and kinetic isotope effects

All the experiments were performed with 1 mM UDP‐GlcA. No significant increase in activity was observed if higher substrate concentration was used.

#### pH studies

For pH optimum studies (at 23 °C), the reaction buffer (50 mm Na_2_HPO_4_, 100 mm NaCl) was prepared in desired pH values (pH 3–9) and BcUGAepi wild‐type was confirmed as stable over this pH range. The pH was adjusted either with 5 mm NaOH or with 10% HCl. UDP‐GlcA (1 mm), NAD^+^ (100 µm) and BcUGAepi (2 µm, 0.07 mg·mL^−1^) were mixed with the buffer, and samples were taken for HPLC analysis at defined time points from 0 to 150 min.

The initial rates (s^−1^) were calculated at each pH value and plotted against the pH. The data for the BcUGAepi wild‐type were fitted to an Eqn (1) where log*k*_cat_ increases with a slope of + 1 below p*K* and is level above p*K*. [42]

(1) $$\log k_{\text{cat}}=\log\left[\frac{C}{1+[H^{+}]/K}\right]$$

where *C* is the pH‐independent value of *k*_cat_, [H^+^] is the proton concentration, and *K* is a proton dissociation constant.

For Y149F variant, the data were fitted using standard linear regression.

#### Kinetic isotope effect measurements

For kinetic isotope effect measurements, BcUGAepi concentration of 0.037 mg·mL^−1^ (1 µm) was used and the reactions (30 µL final volume) were performed in quintuplicates for both UDP‐GlcA and 4‐^2^H‐UDP‐GlcA (1 mm). The substrate concentration used was saturating. The KIE reaction mixtures were quenched (incubation in 50% (v/v) methanol at 90 °C for 5 min) after 5 min and analyzed on HPLC. Initial reaction rates (*V*) were calculated from the linear dependence of the product formed and time used. The KIE was calculated from the ratio of the reaction rates (effectively, *k*_cat_) for the unlabeled and deuterium‐labeled substrate (KIE = *V*_unlabeled_/*V*_labeled_).

### Analytics

#### HPLC

The sugar nucleotides and NAD^+^/NADH were separated with Shimadzu Prominence HPLC‐UV system (Shimadzu, Korneuburg, Austria) on a Kinetex C18 column (5 µm, 100 Å, 50 × 4.6 mm) using an isocratic method with 5% acetonitrile and 95% tetrabutylammonium bromide buffer (40 mm TBAB, 20 mm K_2_HPO_4_/KH_2_PO_4_, pH 5.9) as mobile phase. UDP‐sugars, NAD^+^, and NADH were detected by UV at 262 nm wavelength. The amount of UDP‐GlcA/UDP‐GalA formed was determined based on the relative integrated peak areas and referred to a calibration curve of a commercial standard of UDP‐GlcA.

#### NMR

The purity and identity of the synthesized compounds were determined by ^1^H NMR. All the acquisitions were carried out in D_2_O on a Varian INOVA 500‐MHz NMR spectrometer (Agilent Technologies).

## Conflict of interest

The authors declare no conflict of interest.

## Author contributions

The experimental work included in this paper has been performed by AJEB and HW performed the NMR analyses. AD contributed in designing the synthesis route for UDP‐GalA. BN designed and supervised the research. BN and AJEB wrote the paper.

## Supporting information


**Fig. S1.** Results of SDS‐PAGE of purified BcUGAepi (~37 kDa).
**Fig. S2.** Calibration curve for HiLoad 16/60 Superdex 200 gel filtration column prepared with gel filtration standard mixture #1511901.
**Fig. S3.** Gel filtration chromatogram of BcUGAepi detected by UV absorbance at 280 nm.
**Fig. S4.** Absorbance spectrum of wild‐type BcUGAepi indicating the presence of a protein‐bound nicotinamide cofactor (260 nm, 340 nm).
**Fig. S5.** HPLC chromatograms of NAD^+^, NADP^+^and NADH standards (a‐c) and supernatant of denatured BcUGAepi showing the cofactor content (d).
**Fig. S6.** Influence of NAD+ concentration (0–1000 µM) on the catalytic activity of BcUGAepi with UDP‐GlcA.
**Fig. S7.** Catalytic activity of BcUGAepi in the reaction with UDP‐GlcA at different temperatures.
**Fig. S8.** Anion exchange chromatogram recorded during the purification of UDP‐α‐d‐galacturonic acid (UDP‐GalA).
**Fig. S9.** Chromatogram recorded during the desalting step of UDP‐α‐d‐galacturonic acid.
**Fig. S10.** HPLC chromatogram of purified and desalted UDP‐α‐d‐galacturonic acid. The purity of> 98% was obtained.
**Fig. S11.** ^1^H NMR spectrum (500 MHz, D_2_O) of purified and desalted UDP‐α‐d‐galacturonic acid, δ 5.62 ppm (dd, 1H), 4.41 ppm (s, 1H), 4.23 ppm (m, 1H), 3.92 ppm (dd, 1H), 3.75 ppm (dd, 1H).
**Fig. S12.** Chromatogram recorded during the purification of UDP‐α‐d‐4‐^2^H‐glucuronic acid.
**Fig. S13.** Chromatogram recorded during the desalting of UDP‐α‐d‐4‐^2^H‐glucuronic acid.
**Fig. S14.** HPLC chromatogram of purified and desalted UDP‐α‐D‐4‐2H‐glucuronic acid. The purity of> 99% was obtained.
**Fig. S15.** ^1^H NMR spectrum (500 MHz, D_2_O) of purified and desalted UDP‐α‐d‐4‐^2^H‐glucuronic acid, δ 5.58 ppm (dd, 1H), 4.17 ppm (d, 1H), 3.74 ppm (d, 1H), 3.55 ppm (dd, 1H).
**Fig. S16.** Time course of BcUGAepi reaction with UDP‐GalA as a substrate.
**Fig. S17.** Michaelis‐Menten kinetics of the forward (a. UDP‐GlcA → UDP‐GalA) and reverse (b. UDP‐GalA → UDP‐GlcA) reaction catalyzed by BcUGAepi.
**Fig. S18.** Multiple sequence alignment (prepared with Clustal Omega) of UDP‐galactose 4‐epimerases (GALEs) and BcUGAepi.
**Fig. S19.** Results of SDS‐PAGE of purified BcUGAepi _Y149F (~37 kDa).
**Fig. S20.** Time course of BcUGAepi_Y149F catalyzed reaction with UDP‐GlcA as a substrate.
**Fig. S21.** *In situ* NMR experiments with BcUGAepi.
**Fig. S22.** NADH calibration curve on HPLC.
**Fig. S23.** HPLC chromatograms from denaturation experiments of BcUGAepi reacted with UDP‐GlcA and UDP‐4‐[^2^H]‐GlcA.
**Fig. S24.** Phylogenetic analysis of SDRs active on sugar nucleotides.
**Fig. S25.** (a) A part of a sequence alignment (aligned with Clustal Omega and visualized with ESPript [4]) of UGAepis and GALEs. The residues responsible for glucose binding in GALE [5,6] and the corresponding residues in UGAepis are highlighted in yellow. The amino acids involved in binding of glucose in *E. coli* GALE (structure in panel b) are labelled in red (below the alignment) and the corresponding amino acids in BcUGAepi in blue (above the alignment). UniProt entries: UGAepi BcUGApi (J8BY31), UGAepi UGlcAE (A7GQD3), UGAepi MoeE5 (A0A003), UGAepi Arabidopsis_1 (Q9M0B6), UGAepi Zea mays (Q304Y2), UGAepi Cap1J (P96481), UGAepi GlaKP analog (Q9RP53), GALE E.coli K12 (P09147), GALE Homo sapiens (Q14376), GALE Neiss. gonorr. (Q05026), GALE Salm. typhi (Q56093) and GALE Yersinia pestis (Q9F7D4). (b) Close‐up structure of the active site of GALE (generated with PyMOL) showing the positioning of the conserved glucose‐binding interactions. Residues (light green) responsible for recognition of the sugar moiety: Tyr149, Lys84, Ser124, Asn179, Asn199. Yellow and grey carbon atoms correspond to UDP‐Glc and NADH, respectively. PDB: 1XEL.
**Fig. S26.** (a) Interactions with the carboxylate moiety of UDP‐GlcA (yellow carbons) in the active site of UXS (cyan carbons; PDB: 2B69, sugar modeled into the active site) [7]. (b) Interactions with the carboxylate group in the active site of ArnA (peach carbons; PDB: 1Z7E) [8]. The structures were generated with PyMOL.
**Fig. S27.** Residues (Asn179 and Tyr299) on the active site of GALE (green carbons; PDB: 1XEL) responsible for binding the 6‐OH group of UDP‐Glc (yellow carbons).
**Scheme S1.** NAD^+^dependent oxidative decarboxylation of UDP‐α‐d‐glucuronic acid yielding UDP‐4‐keto‐α‐d‐xylose (and CO_2_) catalyzed by *E. coli* enzyme ArnA.
**Scheme S2.** One‐pot synthesis of UDP‐α‐d‐4‐2H‐glucuronic acid with lactate dehydrogenase‐based regeneration system of NAD+.
