# Supplementary material for: Mechanistic characterization of UDP‐glucuronic acid 4‐epimerase
Source: FEBS J. 2020 Aug 5;288(4):1163–78. doi: 10.1111/febs.15478 (PMC7984243; doi:10.1111/febs.15478)
Supplement: Supplementary file 1 — Fig. S1. Results of SDS‐PAGE of purified BcUGAepi (~37 kDa). Fig. S2. Calibration curve for HiLoad 16/60 Superdex 200 gel filtration column prepared with gel filtration standard mixture #1511901. Fig. S3. Gel filtration chromatogram of BcUGAepi detected by UV absorbance at 280 nm. Fig. S4. Absorbance spectrum of wild‐type BcUGAepi indicating the presence of a protein‐bound nicotinamide cofactor (260 nm, 340 nm). Fig. S5. HPLC chromatograms of NAD+, NADP+and NADH standards (a‐c) and supernatant of denatured BcUGAepi showing the cofactor content (d). Fig. S6. Influence of NAD+ concentration (0–1000 µM) on the catalytic activity of BcUGAepi with UDP‐GlcA. Fig. S7. Catalytic activity of BcUGAepi in the reaction with UDP‐GlcA at different temperatures. Fig. S8. Anion exchange chromatogram recorded during the purification of UDP‐α‐d‐galacturonic acid (UDP‐GalA). Fig. S9. Chromatogram recorded during the desalting step of UDP‐α‐d‐galacturonic acid. Fig. S10. HPLC chromatogram of purified and desalted UDP‐α‐d‐galacturonic acid. The purity of> 98% was obtained. Fig. S11. 1H NMR spectrum (500 MHz, D2O) of purified and desalted UDP‐α‐d‐galacturonic acid, δ 5.62 ppm (dd, 1H), 4.41 ppm (s, 1H), 4.23 ppm (m, 1H), 3.92 ppm (dd, 1H), 3.75 ppm (dd, 1H). Fig. S12. Chromatogram recorded during the purification of UDP‐α‐d‐4‐2H‐glucuronic acid. Fig. S13. Chromatogram recorded during the desalting of UDP‐α‐d‐4‐2H‐glucuronic acid. Fig. S14. HPLC chromatogram of purified and desalted UDP‐α‐D‐4‐2H‐glucuronic acid. The purity of> 99% was obtained. Fig. S15. 1H NMR spectrum (500 MHz, D2O) of purified and desalted UDP‐α‐d‐4‐2H‐glucuronic acid, δ 5.58 ppm (dd, 1H), 4.17 ppm (d, 1H), 3.74 ppm (d, 1H), 3.55 ppm (dd, 1H). Fig. S16. Time course of BcUGAepi reaction with UDP‐GalA as a substrate. Fig. S17. Michaelis‐Menten kinetics of the forward (a. UDP‐GlcA → UDP‐GalA) and reverse (b. UDP‐GalA → UDP‐GlcA) reaction catalyzed by BcUGAepi. Fig. S18. Multiple sequence alignment (prepared with Clustal O [file FEBS-288-1163-s001.zip › febs15478-sup-0001-Supinfo.pdf]

FEBS PRESS
science publishing by scientists
WILEY
The FEBS Journal
www.febsjournal.org

Mechanistic characterization of UDP-glucuronic acid 4-epimerase

Annika J. E. Borg, Alexander Dennig, Hansjörg Weber and Bernd Nidetzky

DOI: 10.1111/febs.15478

SUPPLEMENTARY INFORMATION

Mechanistic characterization of UDP-glucuronic acid 4-epimerase

Annika J. E. Borg[a], Alexander Dennig[a,b], Hansjörg Weber[c] and Bernd Nidetzky[a,b]*

[a] Institute of Biotechnology and Biochemical Engineering, Graz University of Technology, NAWI Graz, Petersgasse 12, 8010 Graz, Austria

[b] Austrian Centre of Industrial Biotechnology, Petersgasse 14, 8010 Graz, Austria

[c] Institute of Organic Chemistry, Graz University of Technology, NAWI Graz, Stremayrgasse 9, 8010 Graz, Austria

* Corresponding author (B.N., bernd.nidetzky@tugraz.at)

DNA sequence of BcUGAepi

ATGAAAATATTAGTAACAGGAGCTGCAGGGTTTATAGGATCACACCTATGTCAAGCGCTGCTGAAG
AACAGCGCGTACCACGTGGTTGGCATTGACCACTTTATTGGTCCGACCCCGGCGACCCTGAAAACC
GGCAACATCCAGAGCCTGGAACTGAACAGCCGTTTCCAATTTATTCGTGAGGACATCCTGAACACC
GATCTGAGCAAACTGCTGCAGGACATTGATGTGGTTTATCATCTGGCGGCGATCCCGGGCGTTCGT
ACCAGCTGGGGCAAGGATTTCCAACCGTATGTGACCAACAACATTATGGTTACCCAGCAACTGCTG
GAAGCGTGCAAGCACATCAAACTGGACAAGTTTATCCACATTAGCACCAGCAGCGTGTACGGCGA
GAAGAGCGGTGCGGTTAGCGAAGATCTGCTGCCGATCCCGCTGAGCCCGTACGGCGTGACCAAAC
TGAGCGGTGAACACCTGTGCCACGTTTATCACAAGAACTTCCACATCCCGATTGTGATCCTGCGTTA
CTTTACCGTTTATGGTCCGCGTCAGCGTCCGGACATGGCGTTCCACCGTCTGATTAAACAAATGCTG
GAGGATAAGCCGCTGACCATCTTCGGTGACGGCACCCAGACCCGTGATTTTACCTATATTGACGAT
TGCATCCGTGGCACCGTGGCGGCGCTGGAAACCAAGAAGAACATCATTGGTGAAGTTATTAACATC
GGTGGCAAAGAGCAAGCGAGCATTCTGGACATCATTAGCATGCTGGAAAAAATCAGCGGCAAGAG
CGCGACCAAAAACTTCCTGAAGAGCGTGCCGGGCGAGCCGAAACAGACCTGGGCGGATATTAGCA
AGGCGAGCACCCTGCTGCAATACAGCCCGACCGTTAGCCTGAGCGATGGCCTGGAGGCGGAATAC
GATTATATCAAACAGCTGTATAAGGGTGACGCGGCGTGGAGCCACCCGCAATTTGAGAAGTAA

Amino acid sequence of BcUGAepi

M K I L V T G A A G F I G S H L C Q A L L K N S A Y H V V G I D H F I G P T P A T L K T G N
I Q S L E L N S R F Q F I R E D I L N T D L S K L L Q D I D V V Y H L A A I P G V R T S W G K
D F Q P Y V T N N I M V T Q Q L L E A C K H I K L D K F I H I S T S S V Y G E K S G A V S E
D L L P I P L S P Y G V T K L S G E H L C H V Y H K N F H I P I V I L R Y F T V Y G P R Q R P
D M A F H R L I K Q M L E D K P L T I F G D G T Q T R D F T Y I D D C I R G T V A A L E T K
K N I I G E V I N I G G K E Q A S I L D I I S M L E K I S G K S A T K N F L K S V P G E P K Q T
W A D I S K A S T L L Q Y S P T V S L S D G L E A E Y D Y I K Q L Y K G D A A W S H P Q F
E K **Stop**

Strep-tag

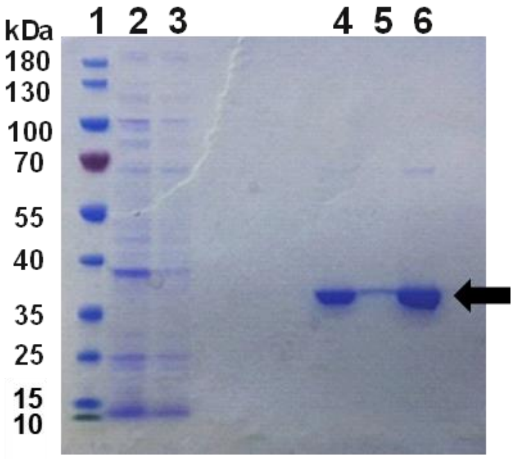

Figure S1. Results of SDS-PAGE of purified BcUGAepi (~37 kDa). Lane 1: molecular mass ladder, lane 2 and 3: flow through fractions from purification, lane 4 and 5: elution fractions from purification, lane 6: concentrated protein sample. Qualitatively identical results were obtained in multiple experiements (N = 10) that included enzyme production, purification and analysis by SDS PAGE as shown.

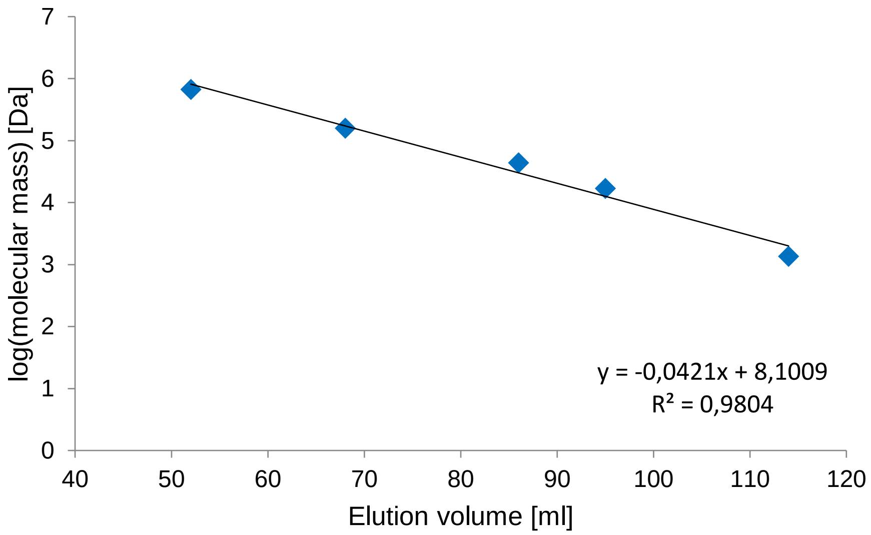

Figure S2. Calibration curve for HiLoad 16/60 Superdex 200 gel filtration column prepared with gel filtration standard mixture #1511901. The logarithm of the molecular masses of thyroglobulin (670000 Da), bovine γ-globulin (158000 Da), chicken ovalbumin (44000 Da), equine myoglobin (17000 Da) and vitamin B12 (1350 Da) is plotted against their elution volumes.

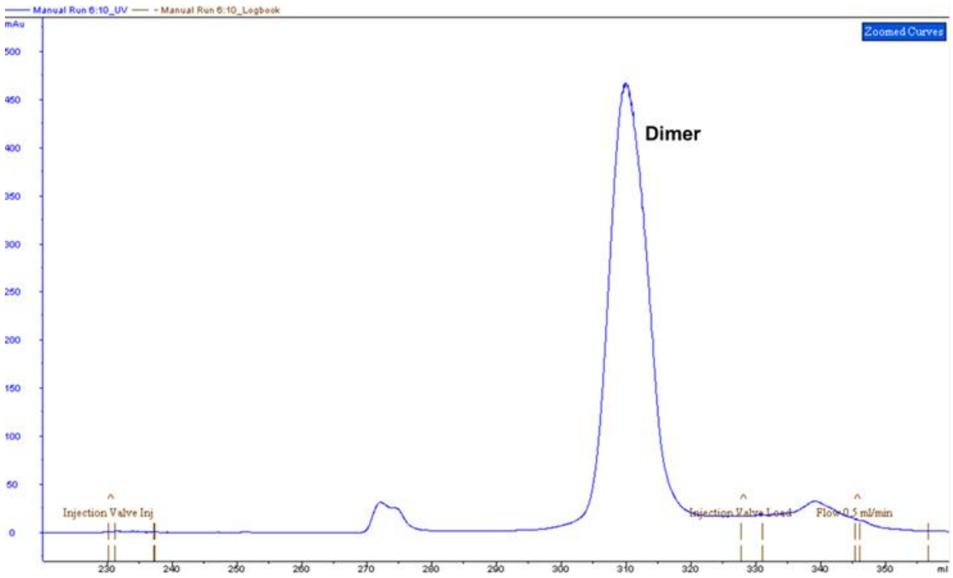

Figure S3. Gel filtration chromatogram of BcUGAepi detected by UV absorbance at 280 nm. The peak corresponding to the apparent molecular mass of BcUGAepi dimer (elution volume 80 ml) is labelled.

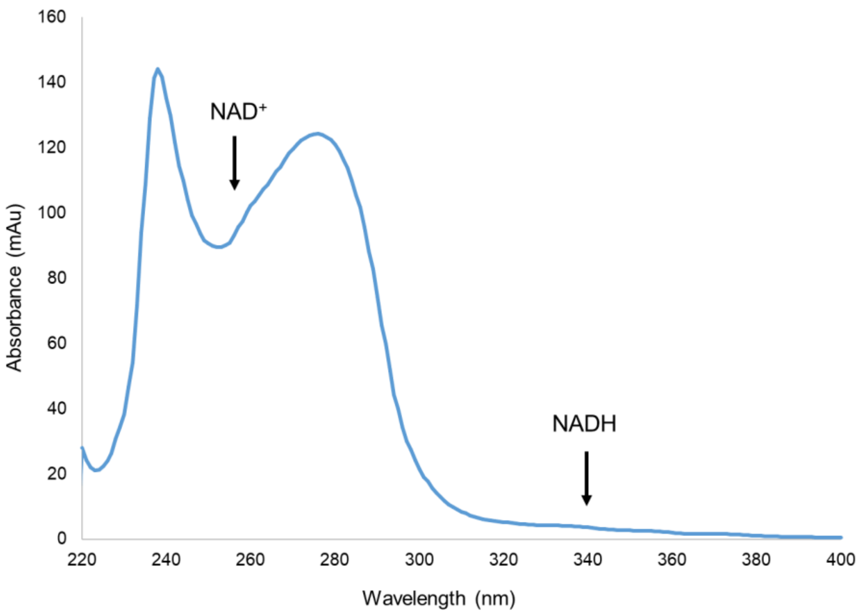

Figure S4. Absorbance spectrum of wild-type BcUGAepi indicating the presence of a protein-bound nicotinamide cofactor (260 nm, 340 nm).

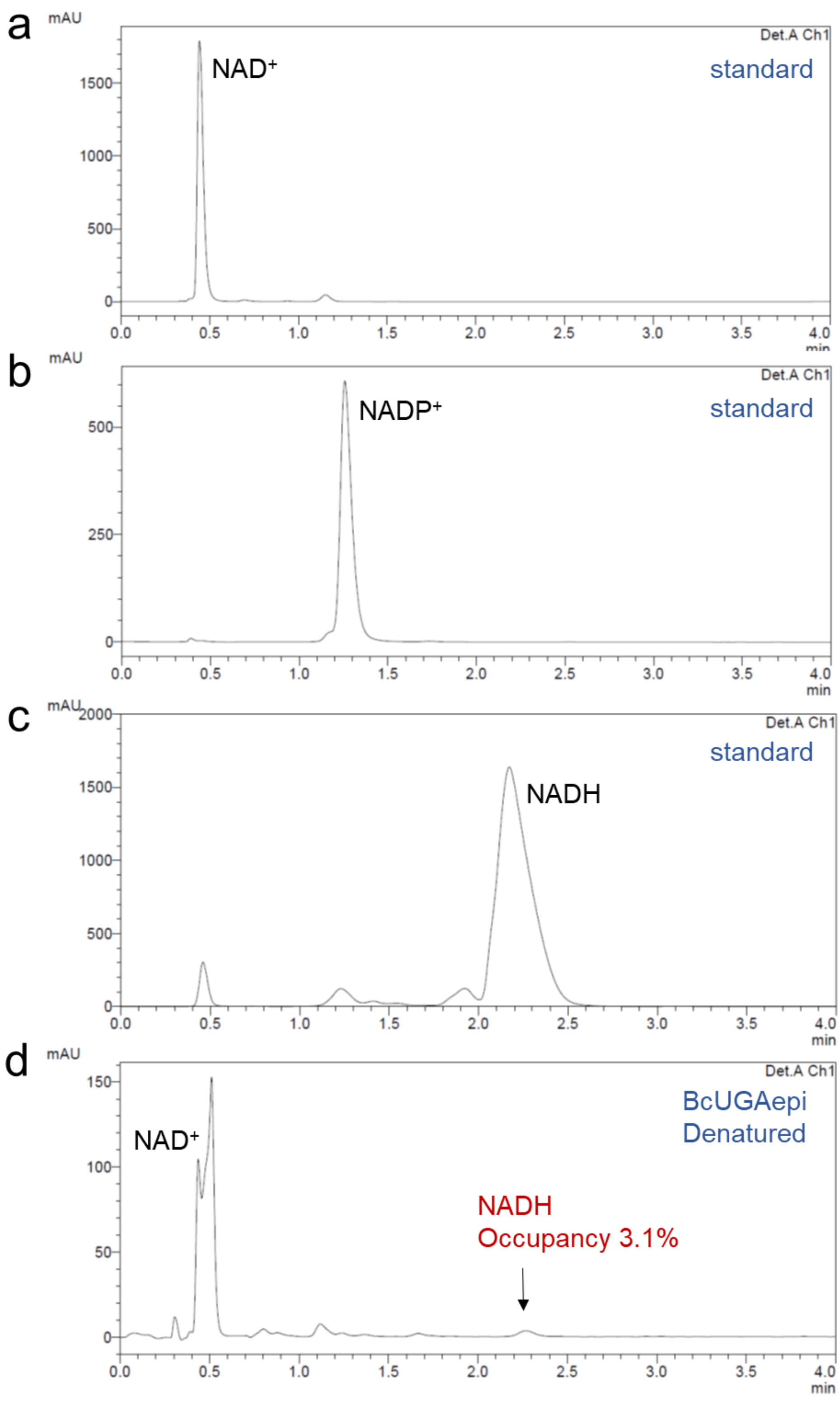

Figure S5. HPLC chromatograms of NAD^+, $NADP^+$ and NADH standards (**a-c**) and supernatant of denatured BcUGAepi showing the cofactor content (**d**).

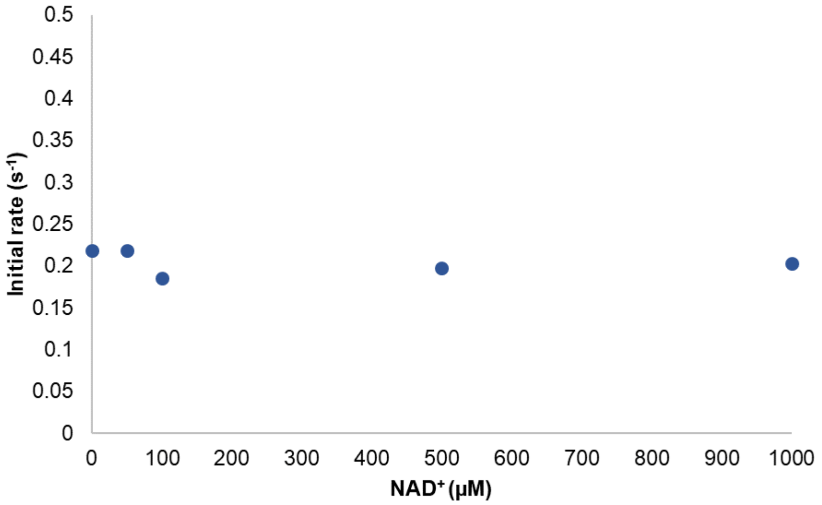

Figure S6. Influence of NAD^+ concentration (0-1000 µM) on the catalytic activity of BcUGAepi with UDP-GlcA. The reactions were performed with 1 mM UDP-GlcA and 1 µM (0.035 mg/ml) purified recombinant BcUGAepi in sodium phosphate buffer (50 mM Na_2HPO_4, 100 mM NaCl, pH 7.6) in final volume of 250 µl. The initial rates (s^{-1}) were plotted against the concentration of NAD^+.

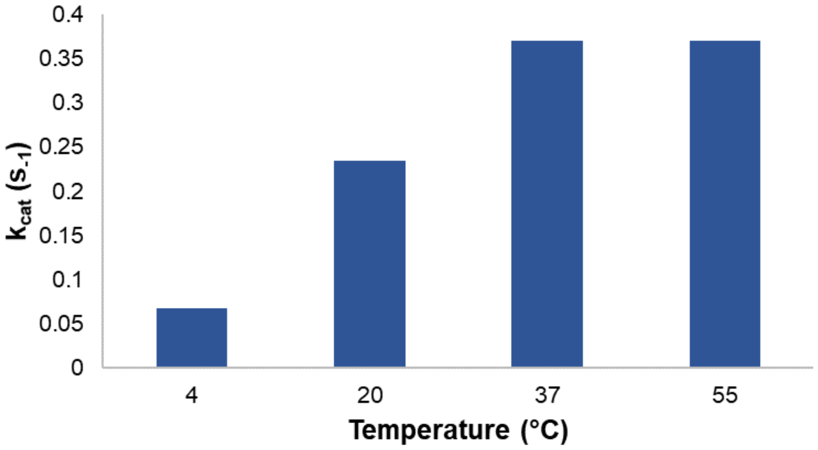

Figure S7. Catalytic activity of BcUGAepi in the reaction with UDP-GlcA at different temperatures. The reactions were performed with 1 mM UDP-GlcA, 100 µM NAD^+ and 2 µM (0.07 mg/ml) purified recombinant BcUGAepi in sodium phosphate buffer (50 mM Na_2HPO_4, 100 mM NaCl, pH 7.6) in final volume of 250 µl. The initial velocities (s^{-1}) were plotted against the temperature.

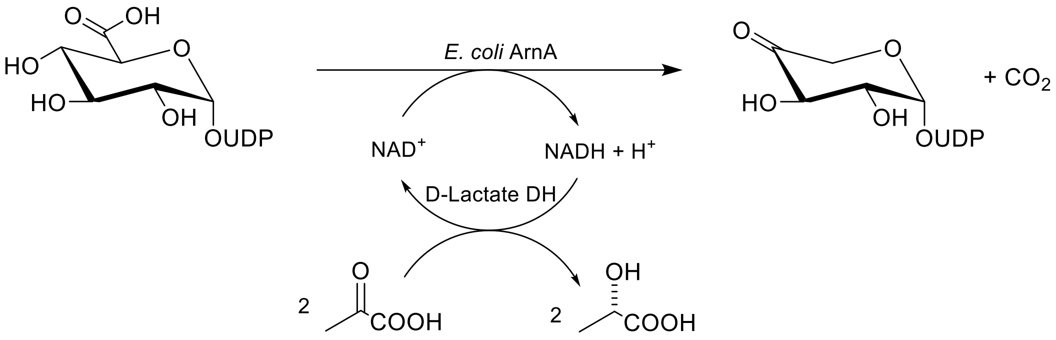

Scheme S1. NAD^+ dependent oxidative decarboxylation of UDP-α-D-glucuronic acid yielding UDP-4-keto-α-D-xylose (and CO_2) catalyzed by *E. coli* enzyme ArnA. Sodium pyruvate (10 mM), 20 U/ml D-lactate dehydrogenase (DH) and 4.7 µM ArnA (0.2 mg/ml) were added into the BcUGAepi reaction mixture and incubated at 30 °C for 16 h until all UDP-GlcA was consumed. UDP = uridine-5'-diphosphate.

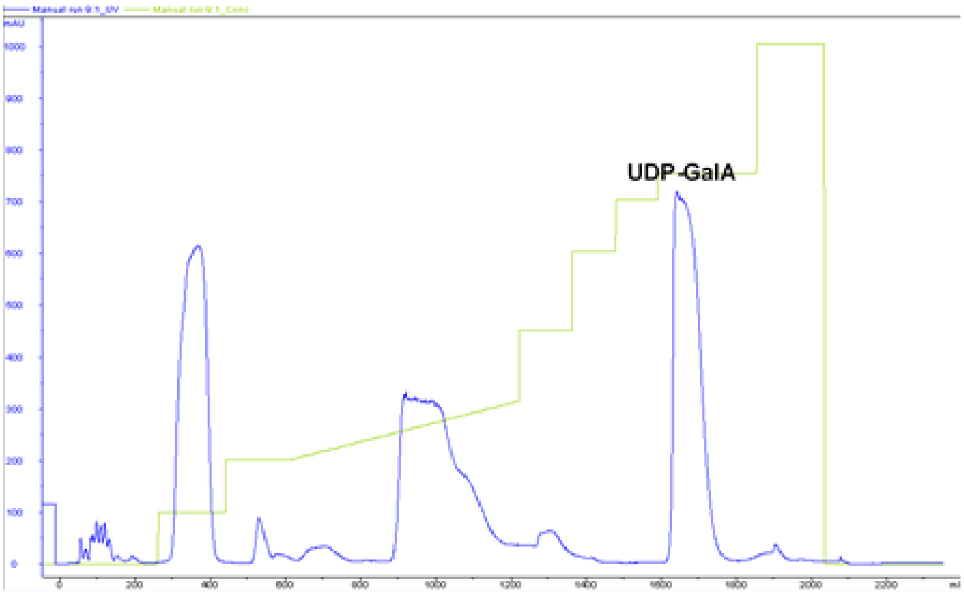

Figure S8. Anion exchange chromatogram recorded during the purification of UDP-α-D-galacturonic acid (UDP-GalA). The UV signal (blue line, 280 nm) of the desired product is labelled as UDP-GalA, the green line corresponds to the salt gradient used for elution. UDP = uridine-5'-diphosphate.

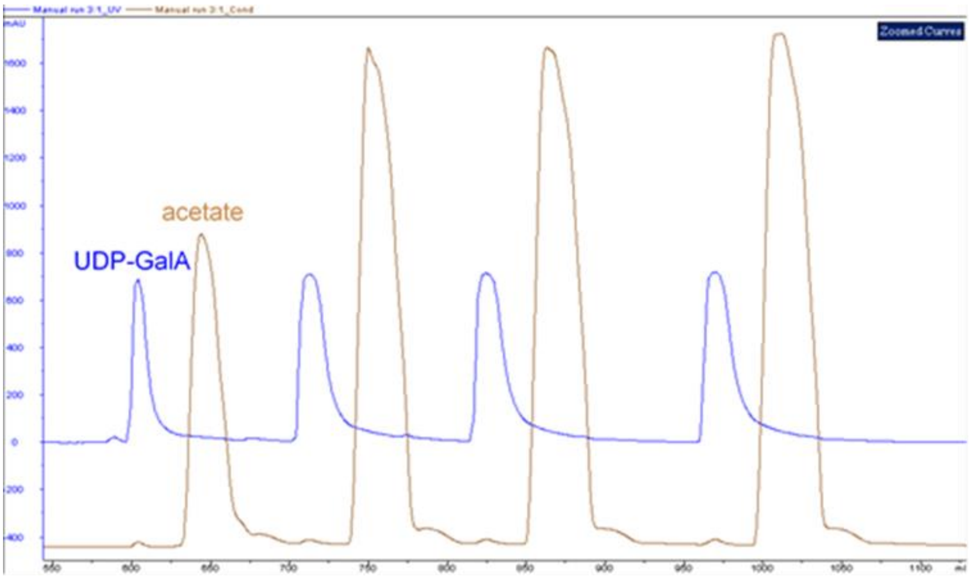

Figure S9. Chromatogram recorded during the desalting step of UDP-α-D-galacturonic acid. The blue line corresponds to the UV-signal of the desired product (at 280 nm), the brown line corresponds to the conductivity signal of acetate.

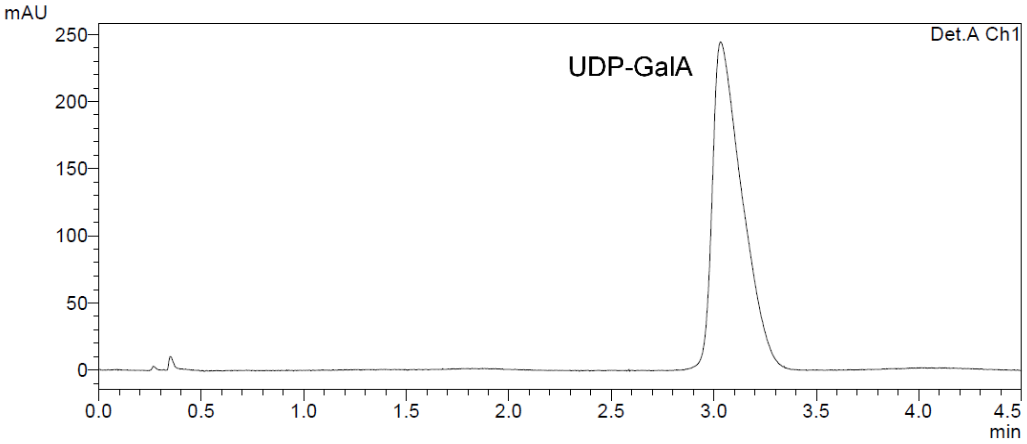

Figure S10. HPLC chromatogram of purified and desalted UDP-α-D-galacturonic acid. The purity of >98% was obtained.

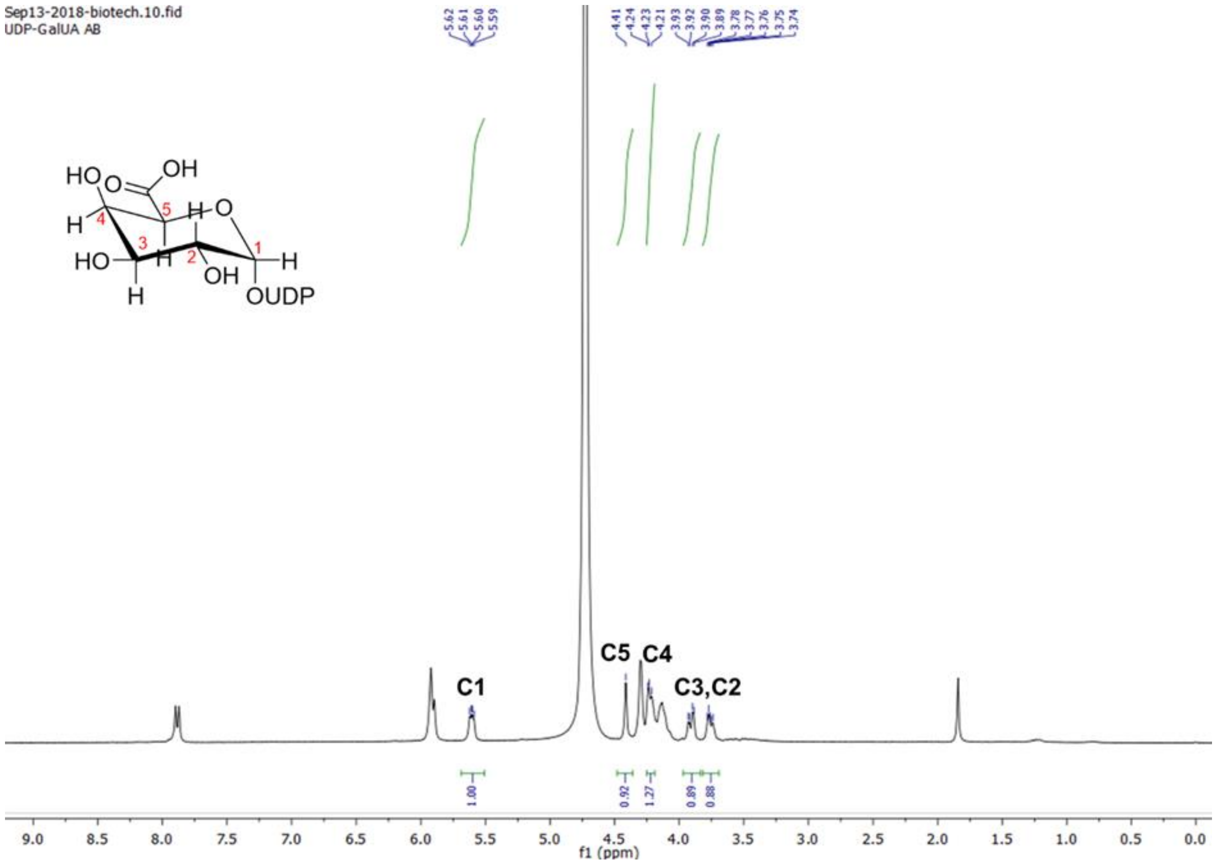

Figure S11. ^{1}H NMR spectrum (500 MHz, D_2O) of purified and desalted UDP-α-D-galacturonic acid, δ 5.62 ppm (dd, 1H), 4.41 ppm (s, 1H), 4.23 ppm (m, 1H), 3.92 ppm (dd, 1H), 3.75 ppm (dd, 1H). The chemical shifts are well in agreement with the values described in literature [1].

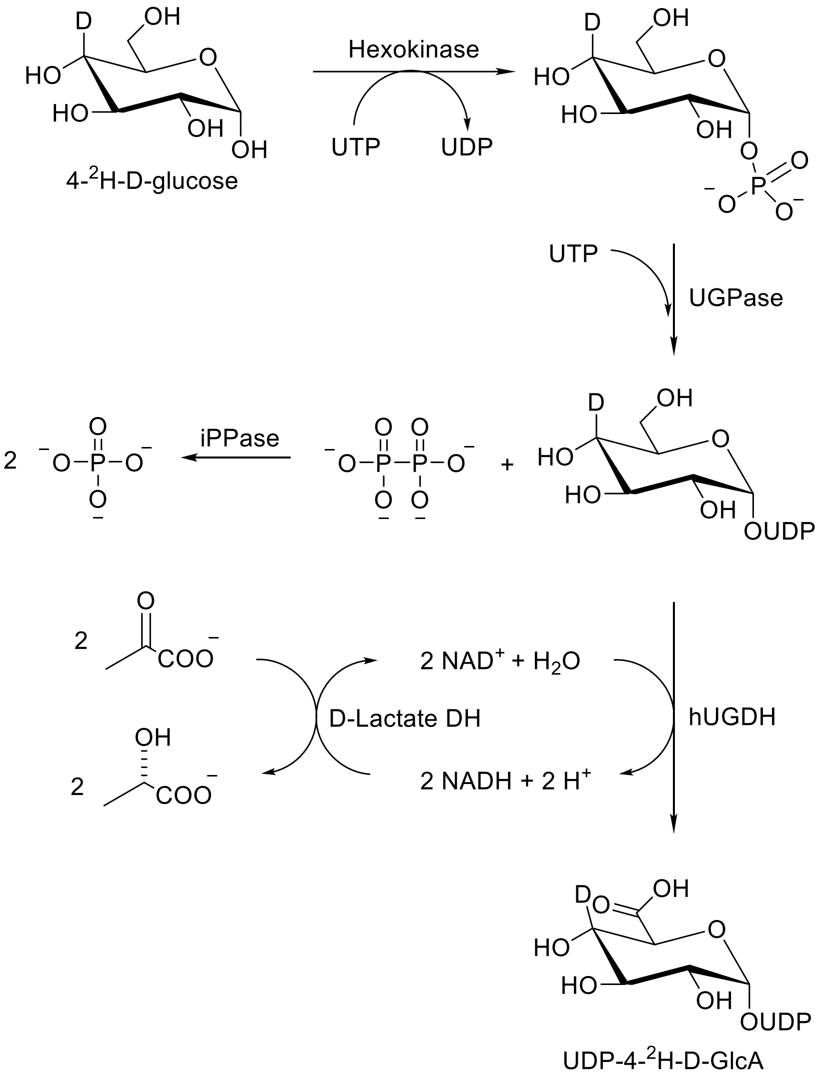

Scheme S2. One-pot synthesis of UDP-α-D-4-^{2}H-glucuronic acid with lactate dehydrogenase-based regeneration system of NAD^+. UTP = uridine-5'-triphosphate, UDP = uridine-5'-diphosphate, UGPase = UDP-glucose pyrophosphorylase, iPPase = inorganic pyrophosphatase, hUGDH = human UDP-glucose 6-dehydrogenase, D-lactate DH = D-lactate dehydrogenase.

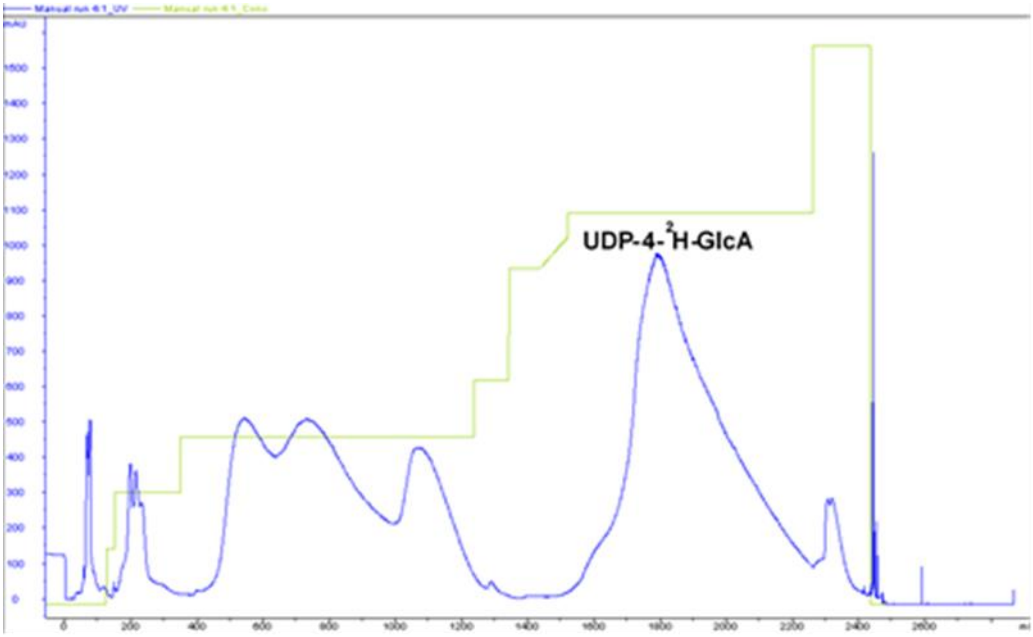

Figure S12. Chromatogram recorded during the purification of UDP-α-D-4-^{2}H-glucuronic acid. The UV peak of the desired product (blue line, 280 nm) is labelled as UDP- 4-^{2}H-GlcA, the green line corresponds to the salt gradient used for elution.

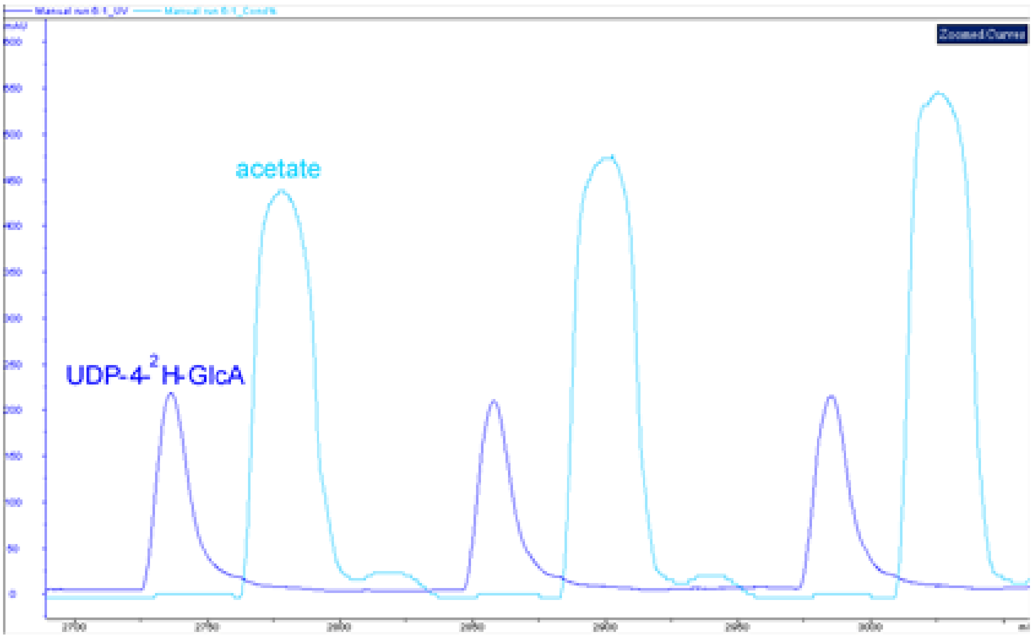

Figure S13. Chromatogram recorded during the desalting of UDP-α-D-4-^{2}H-glucuronic acid. The dark blue line corresponds to the UV-signal of the desired product (at 280 nm), the light blue line shows the conductivity signal of acetate.

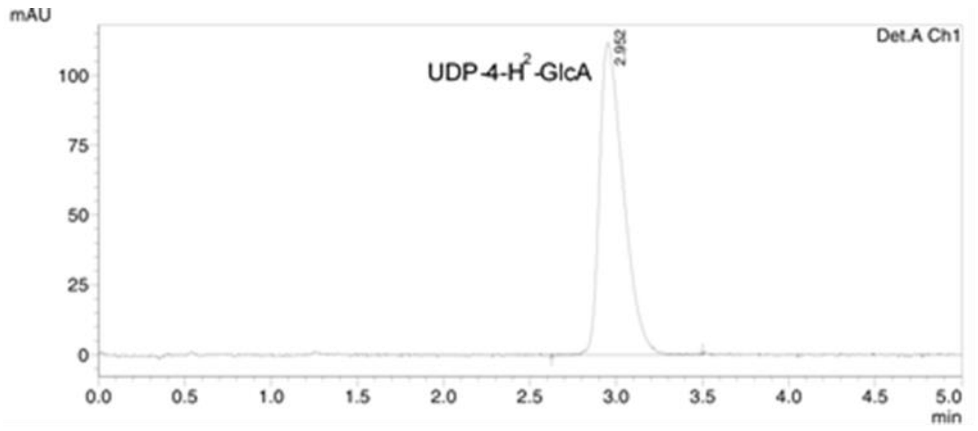

Figure S14. HPLC chromatogram of purified and desalted UDP-α-D-4-^{2}H-glucuronic acid. The purity of >99% was obtained.

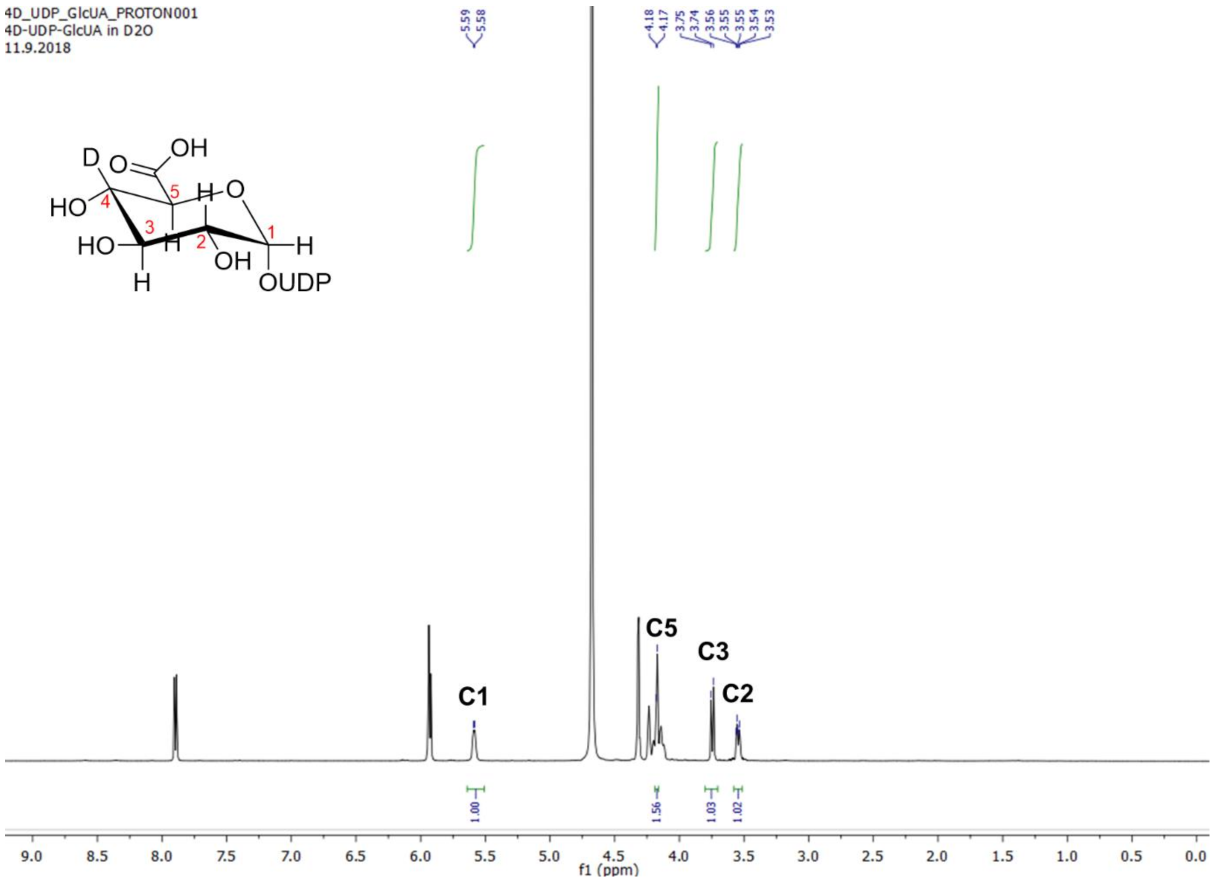

Figure S15. ^{1}H NMR spectrum (500 MHz, D_2O) of purified and desalted UDP-α-D-4-^{2}H-glucuronic acid, δ 5.58 ppm (dd, 1H), 4.17 ppm (d, 1H), 3.74 ppm (d, 1H), 3.55 ppm (dd, 1H).

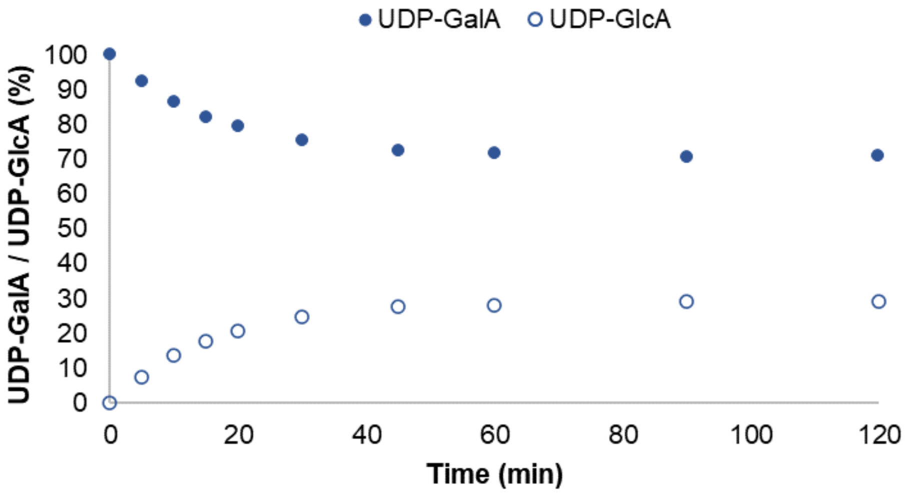

Figure S16. Time course of BcUGAepi reaction with UDP-GalA as a substrate. The reaction mixture contained 1 mM UDP-GalA, 100 µM NAD^+ and 2 µM (0.07 mg/ml) purified recombinant BcUGAepi in sodium phosphate buffer (50 mM Na_2HPO_4, 100 mM NaCl, pH 7.6) in final volume of 250 µl.

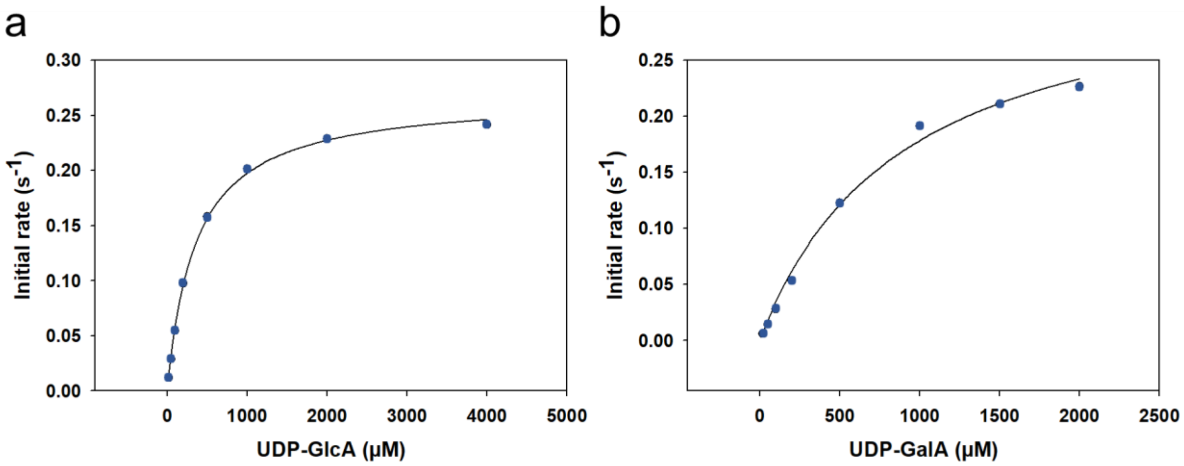

Figure S17. Michaelis-Menten kinetics of the forward (**a.** UDP-GlcA → UDP-GalA) and reverse (**b.** UDP-GalA → UDP-GlcA) reaction catalyzed by BcUGAepi. The reactions contained 2 µM (0.07 mg/ml) BcUGAepi, 50 µM NAD^+ and varying concentration of substrate in sodium phosphate buffer (50 mM Na_2HPO_4, 100 mM NaCl, pH 7.6) in final volume of 50 µl. Each reaction was performed in triplicate and the data was fitted according to the standard Michaelis-Menten kinetic model.

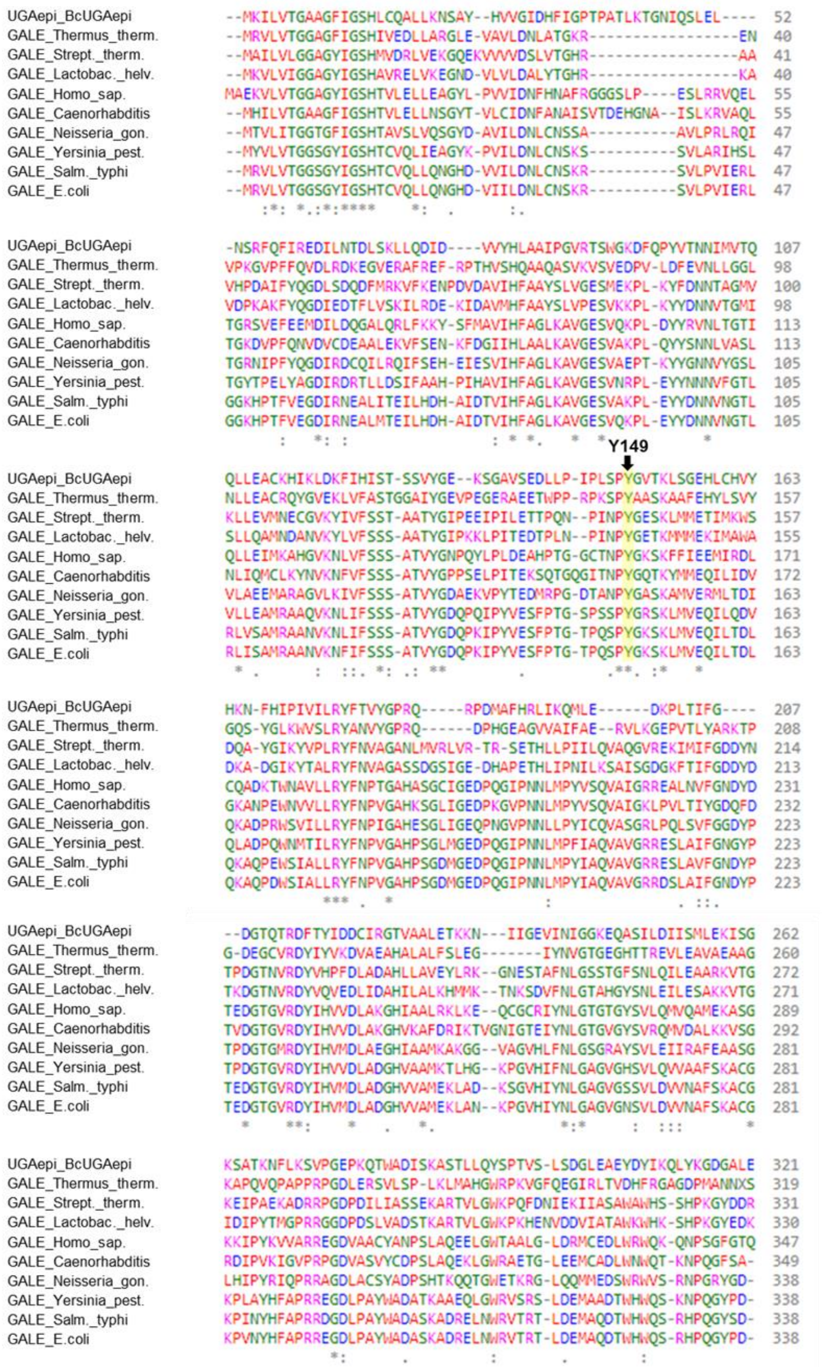

Figure S18. Multiple sequence alignment (prepared with Clustal Omega) of UDP-galactose 4-epimerases (GALEs) and BcUGAepi. Position of the catalytic tyrosine (Tyr149 in BcUGAepi) is highlighted in yellow. UniProt entries: UGAepi_BcUGApi (J8BY31), GALE_Thermus_therm. (Q5SKQ2), GALE_Strept._therm. (P21977), GALE_Lactobac._helv. (Q7WTB1), GALE_Homo_sap. (Q14376), GALE_Caenorhabditis (Q564Q1), GALE_Neisseria_gon. (Q05026), GALE_Yersinia_pest. (Q9F7D4), GALE_Salm._typhi (Q56093) and GALE_E.coli (P09147).

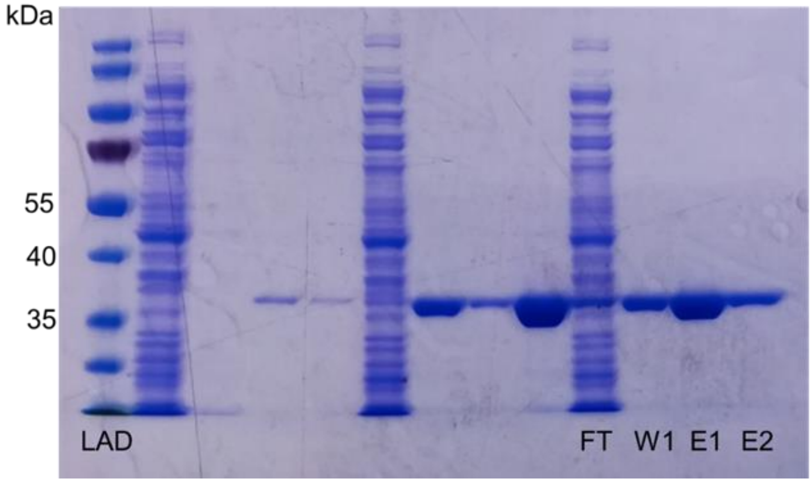

Figure S19. Results of SDS-PAGE of purified BcUGAepi _Y149F (~37 kDa). LAD: molecular mass ladder, FT: flow through (unbound protein), W1: washing fraction from purification, lane E1 and E2: elution fractions from purification. Qualitatively identical results were obtained in multiple experiements (N =3) that included enzyme production, purification and analysis by SDS PAGE as shown.

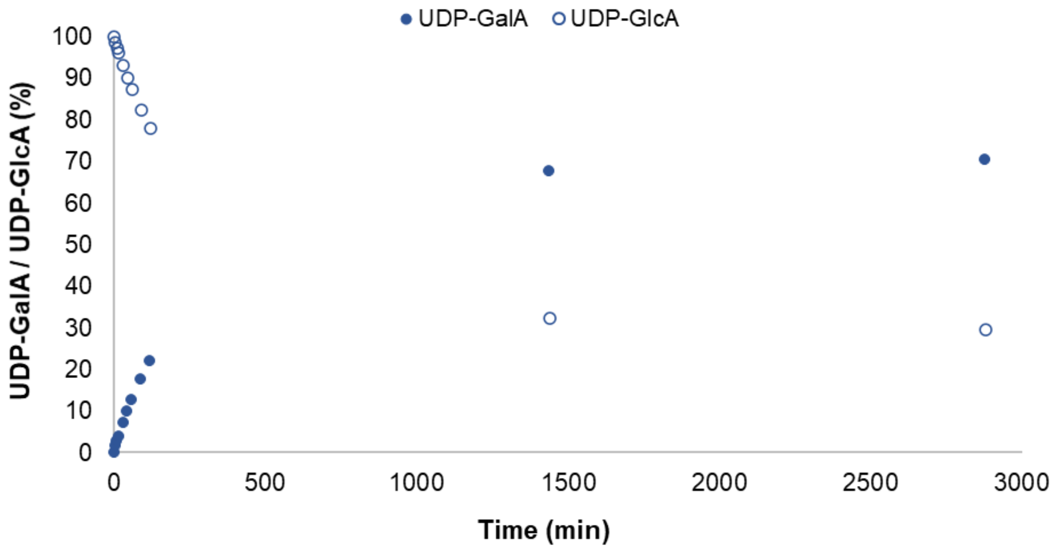

Figure S20. Time course of BcUGAepi_Y149F catalyzed reaction with UDP-GlcA as a substrate. The reaction was performed with 1 mM UDP-GlcA, 100 µM NAD^+ and 135 µM (5 mg/ml) purified recombinant BcUGAepi_Y149F in sodium phosphate buffer (50 mM Na_2HPO_4, 100 mM NaCl, pH 7.6) in final volume of 250 µl. The activity of Y149F variant (0.5 mU/mg) was calculated from the initial velocity (linear part) of the time course.

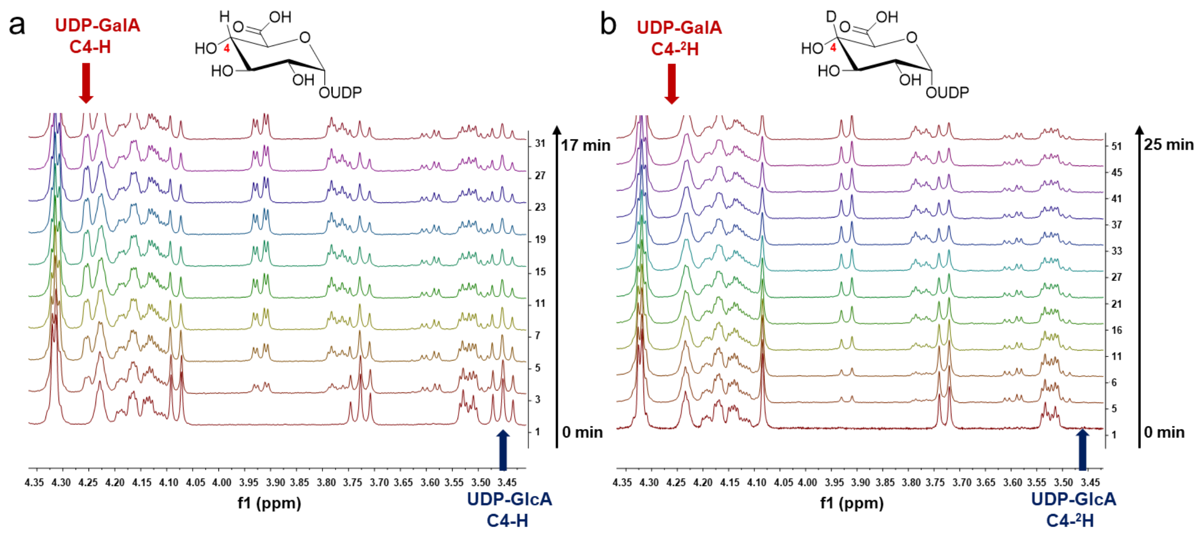

Figure S21. In situ NMR experiments with BcUGAepi. The x-axis shows the chemical shift in ppm and the y-axis the number of the acquisition (only selected spectra are shown for clarity). The direction of the reaction (left: from 0 min in spectrum 1 to 17 min in spectrum 31; right: from 0 min in spectrum 1 to 25 min in spectrum 51) is indicated with an arrow. **a.** Conversion of UDP-GlcA with BcUGAepi. **b.** Conversion of UDP-4-^{2}H-GlcA with BcUGAepi. The missing signals for the C4-H of UDP-GlcA and UDP-GalA are due to the incorporation of deuterium at C4.

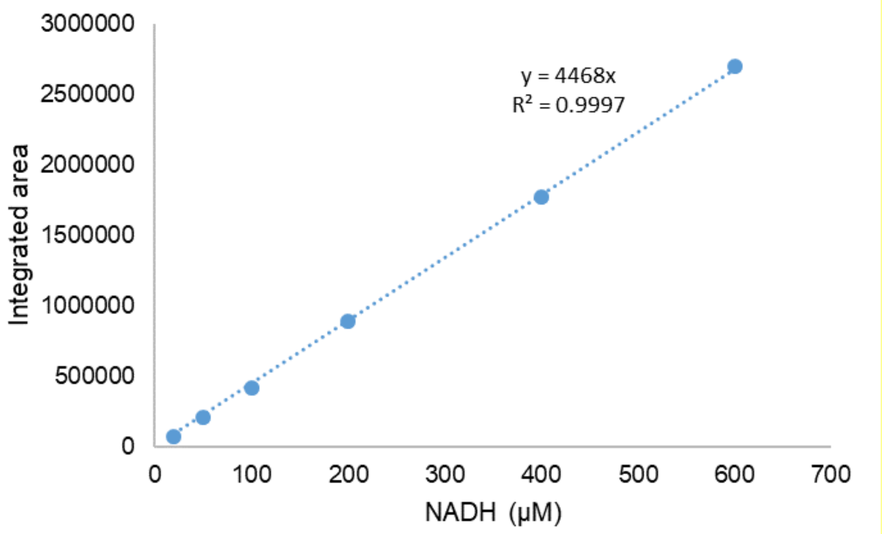

Figure S22. NADH calibration curve on HPLC. The integrated peak areas from HPLC are plotted against the concentration of NADH standards.

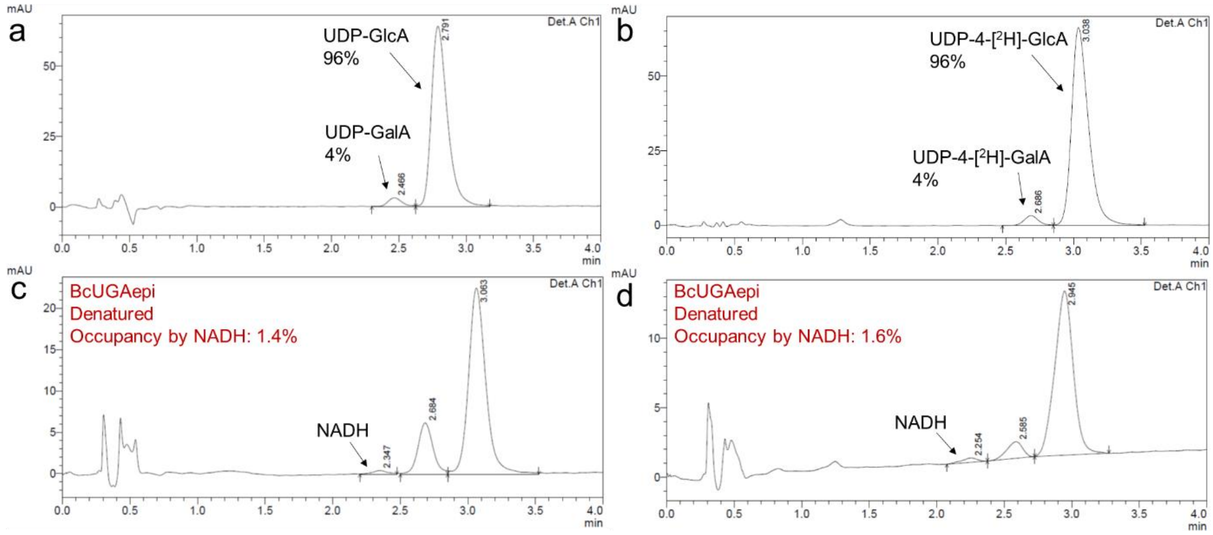

Figure S23. HPLC chromatograms from denaturation experiments of BcUGAepi reacted with UDP-GlcA and UDP-4-[^{2}H]-GlcA. **a,b.** Reaction mixture with UDP-GlcA (**a**) or UDP-4-[^{2}H]-GlcA (**b**) as a substrate at the point where the reaction was stopped and the enzyme filtered out. **c,d.** HPLC chromatograms of the supernatant from the denatured BcUGAepi from reaction a (**c**) and b (**d**).

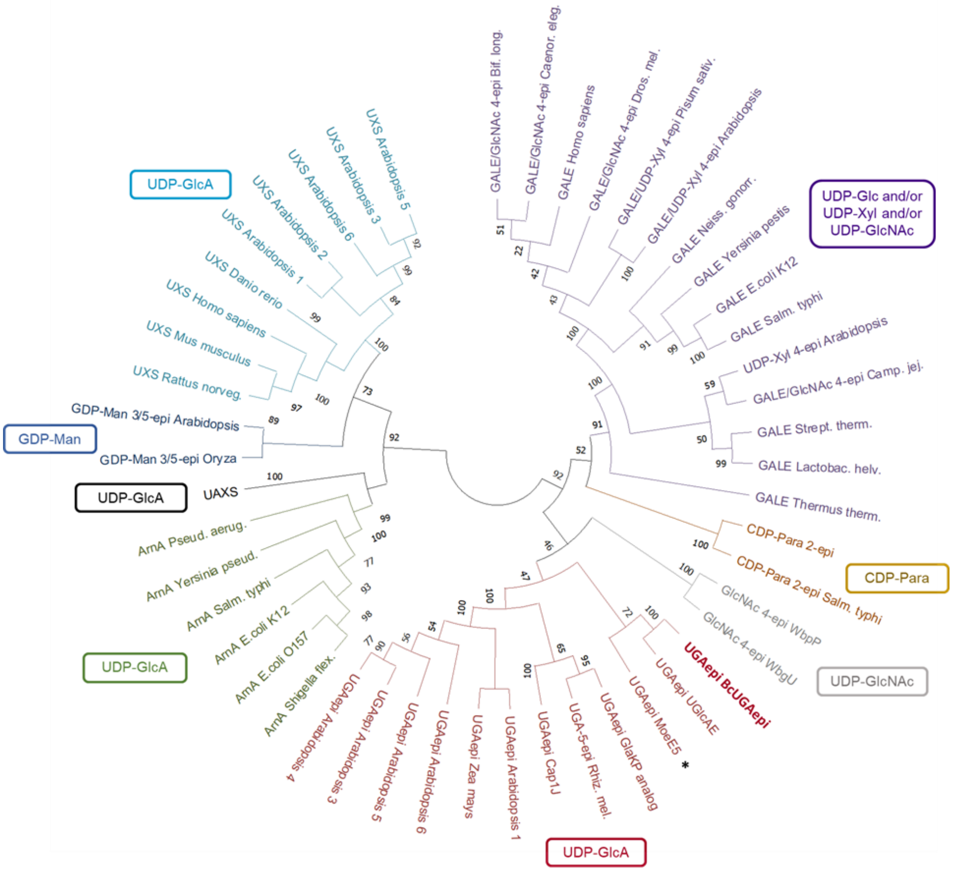

Figure S24. Phylogenetic analysis of SDRs active on sugar nucleotides. Color coding is used to highlight the subgroups of enzymes and their corresponding substrates. The UGAepi with expanded substrate specificity is marked with an asterisk and BcUGAepi is highlighted in bold. The evolutionary history was determined by using the Maximum Likelihood method and Poisson Correction model [2]. The tree with the highest log likelihood is shown and the percentage of trees in which the associated taxa clustered together is shown next to the branches. Initial tree(s) for the heuristic search were obtained automatically by applying Neighbor-Join and BioNJ algorithms to a matrix of pairwise distances estimated using a JTT model, and then selecting the topology with superior log likelihood value. There were a total of 621 positions in the final dataset. Evolutionary analyses were conducted in Mega X [3]. GDP-Man = Guanosine diphosphate mannose, CDP-Para = Cytidine diphosphate paratose, UDP-Glc = Uridine diphosphate glucose, UDP-Xyl = Uridine diphosphate xylose, UDP-GlcNAc = Uridine diphosphate N-acetylglucosamine, UXS = UDP-xylose synthase, UAXS = UDP-apiose/xylose synthase, UGAepi = UDP-glucuronic acid 4-epimerase.

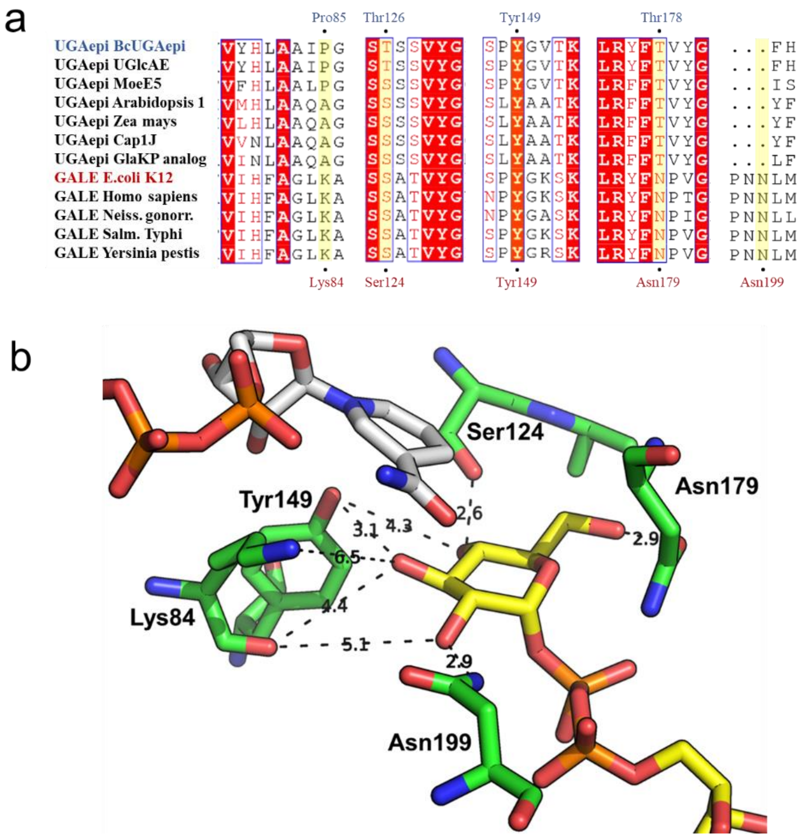

Figure S25. a. A part of a sequence alignment (aligned with Clustal Omega and visualized with ESPript [4]) of UGAepis and GALEs. The residues responsible for glucose binding in GALE [5,6] and the corresponding residues in UGAepis are highlighted in yellow. The amino acids involved in binding of glucose in *E. coli* GALE (structure in panel b) are labelled in red (below the alignment) and the corresponding amino acids in BcUGAepi in blue (above the alignment). UniProt entries: UGAepi BcUGApi (J8BY31), UGAepi UGlcAE (A7GQD3), UGAepi MoeE5 (A0A003), UGAepi Arabidopsis_1 (Q9M0B6), UGAepi Zea mays (Q304Y2), UGAepi Cap1J (P96481), UGAepi GlaKP analog (Q9RP53), GALE E.coli K12 (P09147), GALE Homo sapiens (Q14376), GALE Neiss. gonorr. (Q05026), GALE Salm. typhi (Q56093) and GALE Yersinia pestis (Q9F7D4). **b.** Close-up structure of the active site of GALE (generated with PyMOL) showing the positioning of the conserved glucose-binding interactions. Residues (light green) responsible for recognition of the sugar moiety: Tyr149, Lys84, Ser124, Asn179, Asn199. Yellow and grey carbon atoms correspond to UDP-Glc and NADH, respectively. PDB: 1XEL.

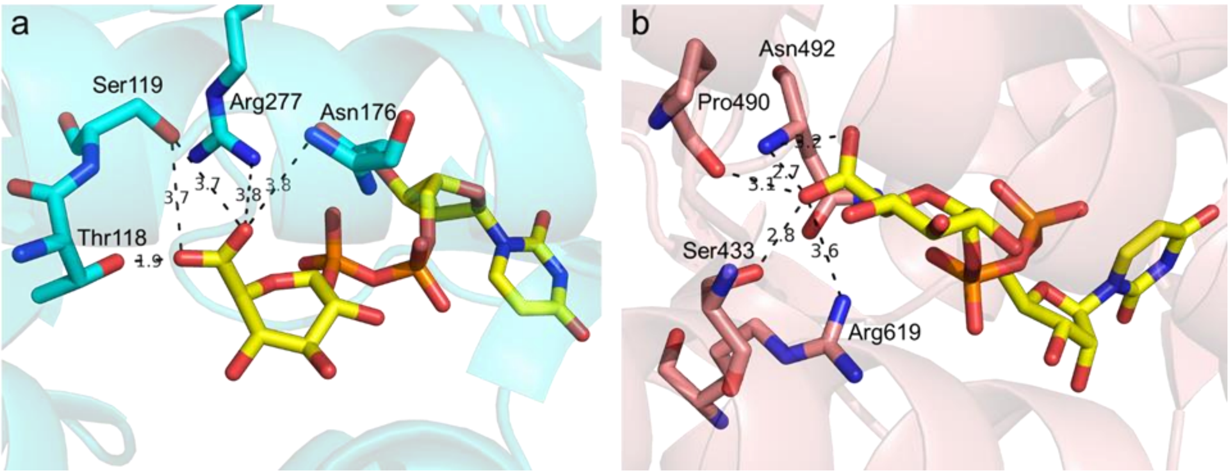

Figure S26. a. Interactions with the carboxylate moiety of UDP-GlcA (yellow carbons) in the active site of UXS (cyan carbons; PDB: 2B69, sugar modeled into the active site) [7]. **b.** Interactions with the carboxylate group in the active site of ArnA (peach carbons; PDB: 1Z7E) [8]. The structures were generated with PyMOL.

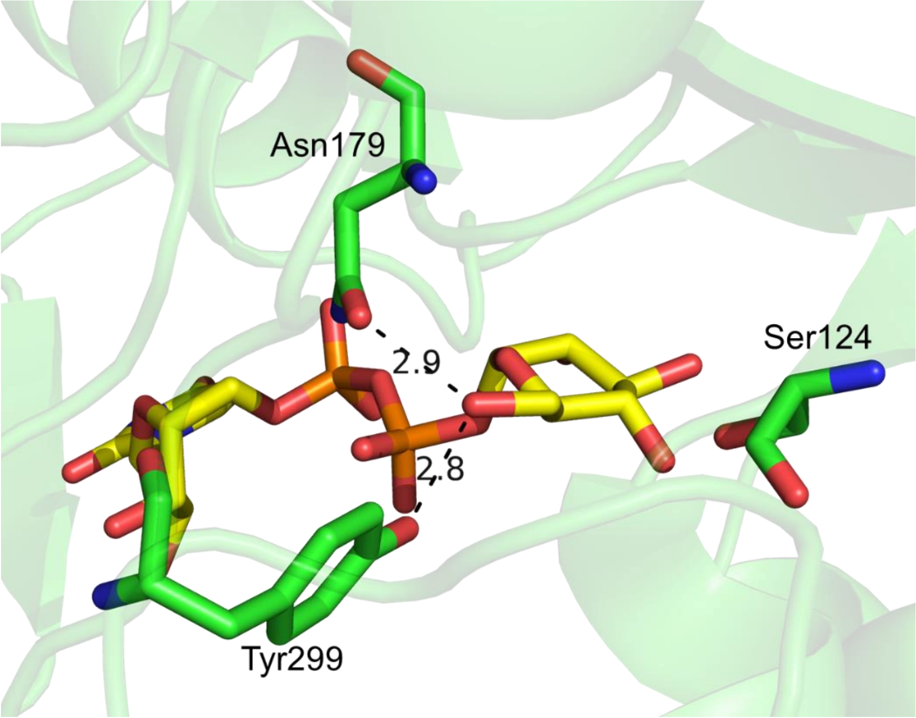

Figure S27. Residues (Asn179 and Tyr299) on the active site of GALE (green carbons; PDB: 1XEL) responsible for binding the 6-OH group of UDP-Glc (yellow carbons). Ser124 (Thr126 in BcUGAepi) is positioned away from 6-OH [5]. PyMOL was used to generate the structure.

Experimental procedures (addition to the main text)

Site-directed mutagenesis

BcUGAepi_Y149F variant was prepared using a modified QuikChange protocol. PCRs were carried out in the reaction volume of 50 µl using 20 ng of plasmid DNA as template and 0.2 µM of forward or reverse primer. Q5 DNA polymerase was used for DNA amplification. The sequences of DNA oligonucleotide primers used for the mutagenesis in BcUGAepi are shown below. The underlined nucleotides highlight the Y149F mutation introduced by PCR.

Name	Mutation	DNA primer sequence (5'-3')
Y149F_fw	Y149F	GATCCCGCTGTCCCCG<u>TTC</u>GGCGTGACCAAACTG
Y149F_rv	Y149F	CAGTTTGGTCACGCC<u>GAA</u>CGGGGACAGCGGGATC

First, three cycles of linear PCR amplification were performed with separate forward and reverse primers (initial denaturation: 30 sec/98 °C; 3 cycles amplification: 10 sec/98 °C for denaturation, 15 sec/55 °C for primer annealing and 6 min/72 °C extension; final extension: 5 min/72 °C). The reaction mixtures were combined to mix the forward and reverse primer solutions, divided again into two mixtures of 50 µl (for better heat transfer) and the PCR program was restarted for 15 cycles of exponential DNA amplification (initial denaturation: 30 sec/98 °C; 3 cycles amplification: 10 sec/98 °C for denaturation, 15 sec/55 °C for primer annealing and 6 min/72 °C extension; final extension: 5 min/72 °C). Residual template DNA was removed by addition of 10 U DpnI and incubation at 37 °C for 16 h. DpnI was inactivated by incubating at 80 °C for 20 min, the mixtures were centrifuged and the PCR products analyzed by agarose gel electrophoresis and visualized by DNA staining. The PCR products were directly transformed into chemically competent *E. coli* NEB5α cells (New England Biolabs). Plasmid DNA was extracted and sequenced with T7prom/T7term primers provided by LGC Genomics (Berlin, Germany) to confirm the mutations. The correct construct was transformed into *E. coli* Lemo21(DE3) cells followed by expression of BcUGAepi as described below.

Expression and purification of BcUGAepi

The synthetic vector pET17b_BcUGAepi was ordered from GenScript (USA) and transformed into chemically competent *E. coli* Lemo21(DE3) cells. BcUGAepi was ordered as a codon optimized gene for optimal expression in *E. coli* and with C-terminal Strep-tag for protein purification (sequence given in the beginning of SI). The cells harboring pET17b_BcUGAepi (or pET17b_BcUGAepi_Y149F) were grown in 10 ml of LB medium (50 μg/ml ampicillin and 35 μg/ml chloramphenicol) at 37 °C for 16 h. From the preculture 2 ml was used to inoculate fresh LB medium (250 ml) supplemented with ampicillin (50 μg/ml) and chloramphenicol (35 μg/ml) and the cells were grown at 37 °C and 120 rpm. After the cell density (OD_{600}) of 0.8 was reached, isopropyl β-D-thiogalactoside (0.2 mM) was added to the culture media to induce gene expression. The cells were incubated at 18 °C and 120 rpm for 20 h. The cells were harvested by centrifugation (2800 g, 4 °C, 20 min), the pellet resuspended in 10 ml of Strep-tag loading buffer (100 mM Tris, 150 mM NaCl, pH 8) and the suspension was stored overnight at -20 °C prior to cell lysis. The cells were disrupted by sonication (pulse 2 sec on, 5 sec off, 70% amplitude, 5 min) and centrifuged (16100 g) at 4 °C for 45 min. The supernatant was collected and filtered (0.45 μm) prior to loading onto the StrepTrap™ HP column (5 ml resin, GE Healthcare Life Sciences) pre-equilibrated with the loading buffer (100 mM Tris, 150 mM NaCl, pH 8). The Strep-tagged protein was eluted with elution buffer (100 mM Tris, 150 mM NaCl, 2.5 mM D-desthiobiotin, pH 8) and re-buffered against reaction buffer (50 mM Na_2HPO_4, 100 mM NaCl, pH 7.6) containing 10% glycerol using Vivaspin filter tubes (30 kDa cut-off). After buffer exchange, the protein was divided into aliquots, flash frozen in liquid nitrogen, and stored at -20 °C. Protein concentration was determined based on the absorption at 280 nm on a Nanodrop spectrophotometer. Size and purity of the protein were confirmed by SDS-PAGE.

Purification and activity assay of UGPase

UGPase was purified by affinity chromatography using a HisTrap 5 ml Ni^{2+} column. The binding buffer was 20 mM imidazole, 100 mM Tris, 50 mM NaCl, pH 7 and the protein was eluted with a buffer containing 400 mM imidazole, 100 mM Tris, 50 mM NaCl, pH 7. Activity was measured in 50 mM Tris-HCl buffer (pH 7.5) supplemented with 5 mM $MgCl_2$, 15 mM UTP, 0.13% (w/v) BSA, 5 mM glucose 1-phosphate and traces of glucose 1,6-bisphosphate. The reactions were started by addition of UGPase in various concentrations (0.064 mg/ml, 0.16 mg/ml, 0.32 mg/ml and 0.64 mg/ml). Samples were taken (0, 5, 10, 15, 30, 60 min) and the reaction quenched by addition of acetonitrile up to 25% (v/v) final concentration prior to HPLC analysis. The increasing concentration of UDP-glucose was plotted against time and the specific activity of UGPase was calculated from the slope of a linear fit to that curve by using equation 1, where k is the slope and c_{enzyme} corresponds to the enzyme concentration.

$$Specific\ activity[\frac{U}{mg}] = \frac{k[mmol/(min \times l)]}{c_{enzyme}[mg/ml]} \qquad (1)$$

One unit of UGPase activity is defined as the amount of enzyme producing 1 μmol of UDP-glucose from UTP and glucose 1-phosphate per minute.

Anion exchange chromatography of sugar nucleotides

UDP-α-D-galacturonic acid

A step-wise gradient of 1 M sodium acetate buffer (pH 4.3) was used to elute the compounds bound to the column. The steps were: 270 ml of 20 mM NaOAc, 160 ml of 100 mM NaOAc, 170 ml of 200 mM NaOAc, 620 ml of NaOAc from 200 to 300 mM with gradient for 140 min, 160 ml of 450 mM NaOAc, 140 ml of 600 mM NaOAc, 140 ml of 700 mM NaOAc, 250 ml of 800 mM NaOAc, 200 ml of 1000 mM NaOAc, 300 ml of 20 mM NaOAc.

UDP-α-D-4-^{2}H-glucuronic acid

During the anion exchange chromatography following flow settings were used: 120 ml of 20 mM NaOAc, 220 ml of 200 mM NaOAc, 900 ml of 300 mM NaOAc, 100 ml of 400 mM NaOAc, 100 ml of 600 mM NaOAc, 120 ml of NaOAc from 600 to 670 mM with gradient for 30 min, 680 ml of 700 mM NaOAc, 200 ml of 1000 mM NaOAc, 200 ml of 20 mM NaOAc.

Oligomeric state of BcUGAepi

For determining the oligomeric state of BcUGAepi, gel filtration was performed with an ÄKTA FPLC connected to HiLoad 16/6 Superdex 200 prep grade column (GE Healthcare) and 1 ml sample loop. The protein was eluted with 150 mM K_2HPO_4 buffer (pH 7.6) (Figure S3) and the apparent molecular mass was calculated from a calibration curve (Figure S2) which was prepared with gel filtration standard mixture #1511901 (Bio-Rad).

References

1. Gu X & Bar-Peled M (2004) The biosynthesis of UDP-galacturonic acid in plants. Functional cloning and characterization of *Arabidopsis* UDP-D-glucuronic acid 4-epimerase. *Plant Physiol* 136, 4256–64.
2. Zuckerkandl E & Pauling L (1965) Evolutionary divergence and convergence in proteins in *Evolving Genes and Proteins* pp. 97–166. Elsevier.
3. Kumar S, Stecher G, Li M, Knyaz C & Tamura K (2018) MEGA X: Molecular evolutionary genetics analysis across computing platforms. *Mol Biol Evol* 35, 1547–1549.
4. Robert X & Gouet P (2014) Deciphering key features in protein structures with the new ENDscript server. *Nucleic Acids Res* 42, W320–324.
5. Thoden JB, Frey PA & Holden HM (1996) Molecular structure of the NADH/UDP-glucose abortive complex of UDP-galactose 4-epimerase from *Escherichia coli*: Implications for the catalytic mechanism. *Biochemistry* 35, 5137–5134.
6. Nam YW, Nishimoto M, Arakawa T, Kitaoka M & Fushinobu S (2019) Structural basis for broad substrate specificity of UDP-glucose 4-epimerase in the human milk oligosaccharide catabolic pathway of *Bifidobacterium longum*. *Sci Rep* 9, 11081.
7. Eixelsberger T *et al.* (2012) Structure and mechanism of human UDP-xylose synthase. *J Biol Chem* 287, 31349–31358.
8. Gatzeva-Topalova PZ, May AP & Sousa MC (2005) Structure and mechanism of ArnA: conformational change implies ordered dehydrogenase mechanism in key enzyme for polymyxin resistance. *Structure* 13, 929–942.
